# New Insights Into Biomphalysin Gene Family Diversification in the Vector Snail *Biomphalaria glabrata*

**DOI:** 10.3389/fimmu.2021.635131

**Published:** 2021-04-01

**Authors:** Silvain Pinaud, Guillaume Tetreau, Pierre Poteaux, Richard Galinier, Cristian Chaparro, Damien Lassalle, Anaïs Portet, Elodie Simphor, Benjamin Gourbal, David Duval

**Affiliations:** ^1^IHPE, Univ Montpellier, CNRS, IFREMER, Univ Perpignan Via Domitia, Perpignan, France; ^2^CNRS, IFREMER, University of Montpellier, Perpignan, France

**Keywords:** biomphalaria, biomphalysin, aerolysin, invertebrate immunity, pore-forming toxin (PFT), structure

## Abstract

Aerolysins initially characterized as virulence factors in bacteria are increasingly found in massive genome and transcriptome sequencing data from metazoans. Horizontal gene transfer has been demonstrated as the main way of aerolysin-related toxins acquisition in metazoans. However, only few studies have focused on their potential biological functions in such organisms. Herein, we present an extensive characterization of a multigene family encoding aerolysins - named biomphalysin - in *Biomphalaria glabrata* snail, the intermediate host of the trematode *Schistosoma mansoni*. Our results highlight that duplication and domestication of an acquired bacterial toxin gene in the snail genome result in the acquisition of a novel and diversified toxin family. Twenty-three biomphalysin genes were identified. All are expressed and exhibited a tissue-specific expression pattern. An *in silico* structural analysis was performed to highlight the central role played by two distinct domains i) a large lobe involved in the lytic function of these snail toxins which constrained their evolution and ii) a small lobe which is structurally variable between biomphalysin toxins and that matched to various functional domains involved in moiety recognition of targets cells. A functional approach suggests that the repertoire of biomphalysins that bind to pathogens, depends on the type of pathogen encountered. These results underline a neo-and sub-functionalization of the biomphalysin toxins, which have the potential to increase the range of effectors in the snail’s immune arsenal.

## Introduction

A diverse array of toxins widely distributed among organisms has emerged as a key factor involved in virulence or defense factors of host/pathogen interactions (1–6). Accordingly, toxins can be found in a wide variety of organisms from bacteria to metazoans and exhibit a highly specific role on a broad range of cellular pathways, thereby triggering a wide variety of physiological effects such as neurotoxicity or cytolysis/necrosis (7–11). Selective pressures operating in host-pathogen interactions have generated a large repertoire of specific toxins that harbor a distinct selectivity (12–16). Among the most common toxins present in all kingdoms, Pore-Forming Toxins (PFT) are a large class of biological weapons used by prokaryotes as virulence factors and by eukaryotes in defense responses or predation functions (17, 18). Overall, these toxins are secreted as soluble protoxins and undergo a conformational change to form oligomeric pores in membranes subsequent to the interaction with specific receptors located at the membrane surface of targeted cells (19–21). In some cases, proteolytic cleavages are required in order to form the active toxin (22–25). Many studies have highlighted the extensive range of residues recognized by PFTs, which include: i) lipids, ii) sugars or iii) membrane proteins (20, 26–29). However, only a few targets have been identified so far for PFTs. Proteins present on immune cell membranes like CCR and CXR (chemokine receptors) or the C5a receptor, the LPS-induced TNF-α factor (LITAF), the CD59 or HAVCR1 (hepatitis A virus cellular receptor) targeted by leukocidins (30, 31), intermedilysin (32), ε-toxin (33) or hemolysin BL (34) respectively, are well-established.

Among PFTs, several members of the aerolysin family, which derive their name from the bacterium genus *Aeromonas*, have been identified in many metazoan genomes as inherited by horizontal gene transfer (HGT) (18, 35). In plants, production of cytolytic enterolobin in seeds seems to provide protection from herbivore grazing and/or insect attack (36). In Cnidaria, hydralysin and nylysin-1b are thought to play a role in host defense against predators and for killing prey (35, 37). In vertebrates, Dln1/Aep1 which shows a high affinity for mannose glycans is proposed to be an immune defense molecule for the zebrafish *Danio rerio* (38, 39) and βγ-CAT protects the frog *Bombina maxima* against the pathogenic bacterium, *Comamonas* sp. (40). Interestingly, this aerolysin-like toxin harbors other non-immune functions such as that involved in tissue repair (41), highlighting the diversified functions that these toxins may have. Another aerolysin-related protein, named biomphalysin, characterized in the Schistosomiasis vector snail *Biomphalaria glabrata* was proposed to have been acquired by HGT (42). Phylogenetic incongruence between aerolysin-related gene trees and species phylogeny revealed that intronless biomphalysin gene clustered with genes from a cnidarian *N. vectensis* and Gram-negative bacteria (42, 43). When produced recombinantly, this toxin was shown to bind to the membranes of the metazoan parasite *Schistosoma mansoni* and to participate in its elimination through cytolysis (42). In addition, a genome-wide study revealed that the biomphalysin locus was strongly correlated with resistance to *S. mansoni* (44) and a transcriptomic analysis indicated that re-exposure to *S. mansoni* triggered an increased representation of biomphalysin transcripts and protein release in the hemolymph of *B. glabrata* snails (45).

In this study, we report the expansion of this Biomphalysin family. Interestingly, data from the released genome of *B. glabrata* provide evidence for the presence of multiple loci coding for biomphalysins in the snail (45, 46). We have focused on the comprehensive characterization of the expansion of this β-PFT biomphalysin family by combining genomic, transcriptomic, structural and functional analyses in *B. glabrata*. Together, our results provide evidence that these toxin genes were diversified and used as potential weapons in the snail’s immune defense arsenal.

## Methods

### Phylogenetic Analysis

**Figure 1:**
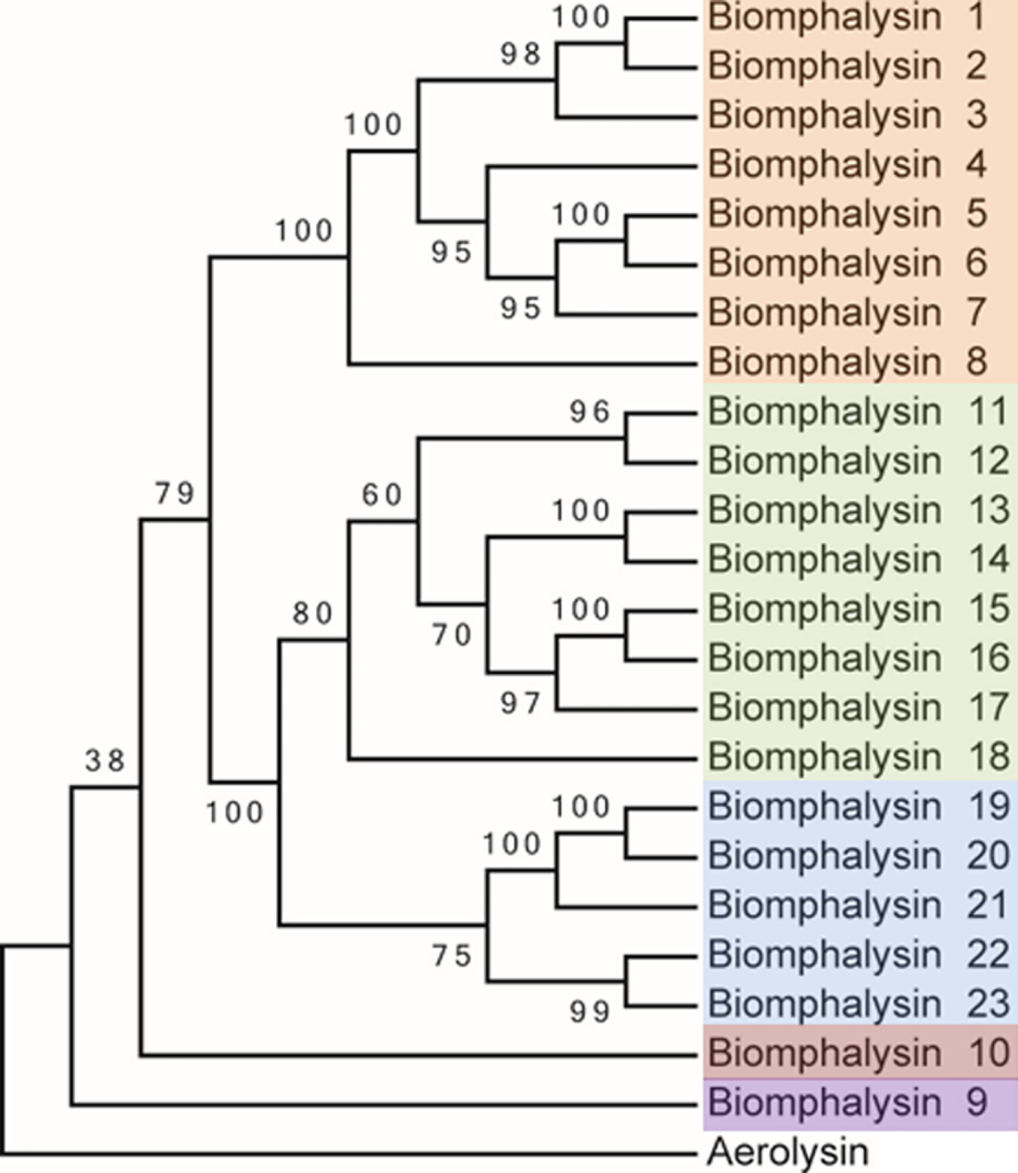
Phylogenetic analysis of the protein sequences from the 23 biomphalysins from *Biomphalaria glabrata*. The three clusters identified in this analysis are highlighted in orange, green and blue for the clusters I, II and III that contain biomphalysins 1 to 8, 11 to 18 and 19 to 23, respectively. Biomphalysin 9 in purple and 10 in red do not group within biomphalysin clusters. The aerolysin sequence from *Aeromonas hydrophila* (GenBank P09167) was used as an outgroup. aLRT was carried out on the tree inferred from the Maximum Likelihood method. Ratio test values multiplied by 100 are shown at each branch of the trees. The accession number of each gene and protein used is listed in **Table S1**.

The 23 biomphalysin protein sequences were retrieved from the genome of *B. glabrata*. To rule out eventual errors in genome assembly, PCRs were performed on snail genomic DNA using specific primer pairs of the predicted coding DNA sequence of each biomphalysin gene (**Table S2**). The protein sequence (GenBank ACC Number: P09167) corresponding to the crystallized aerolysin from *Aeromonas hydrophila* was used as outgroup. Sequences were aligned using Clustal Omega from the Seaview software version 5.0.4 (47, 48). The alignment was then subjected to Gblocks program (49) with the less stringent parameters to trim the non-aligned amino acids. Then we used IQ-TREE v1.6.12 (50), an online phylogenetic tool (http://www.iqtree.org/) to determine the best model for a maximum likelihood analysis using the Bayesian information criterion (BIC). The phylogenetic tree was further generated using the Maximum Likelihood method from PhyML v3.0 software (51), with the following parameters (model: WAG/Amino acid equilibrium frequencies: empirical/Proportion of Invariable site: estimated/Gamma distribution parameter: estimated/Number of substitution rate categories: 4/Tree topology search: NNIs/Starting tree: BioNJ). Approximate Likelihood-Ratio Test (aLRT) was carried out to assess the robustness of the branches on the trees inferred from the maximum likelihood method. Values are shown on each branch of the trees generated. Tree is only presented in topology format (**Figure 1**).

**Figure 2:**
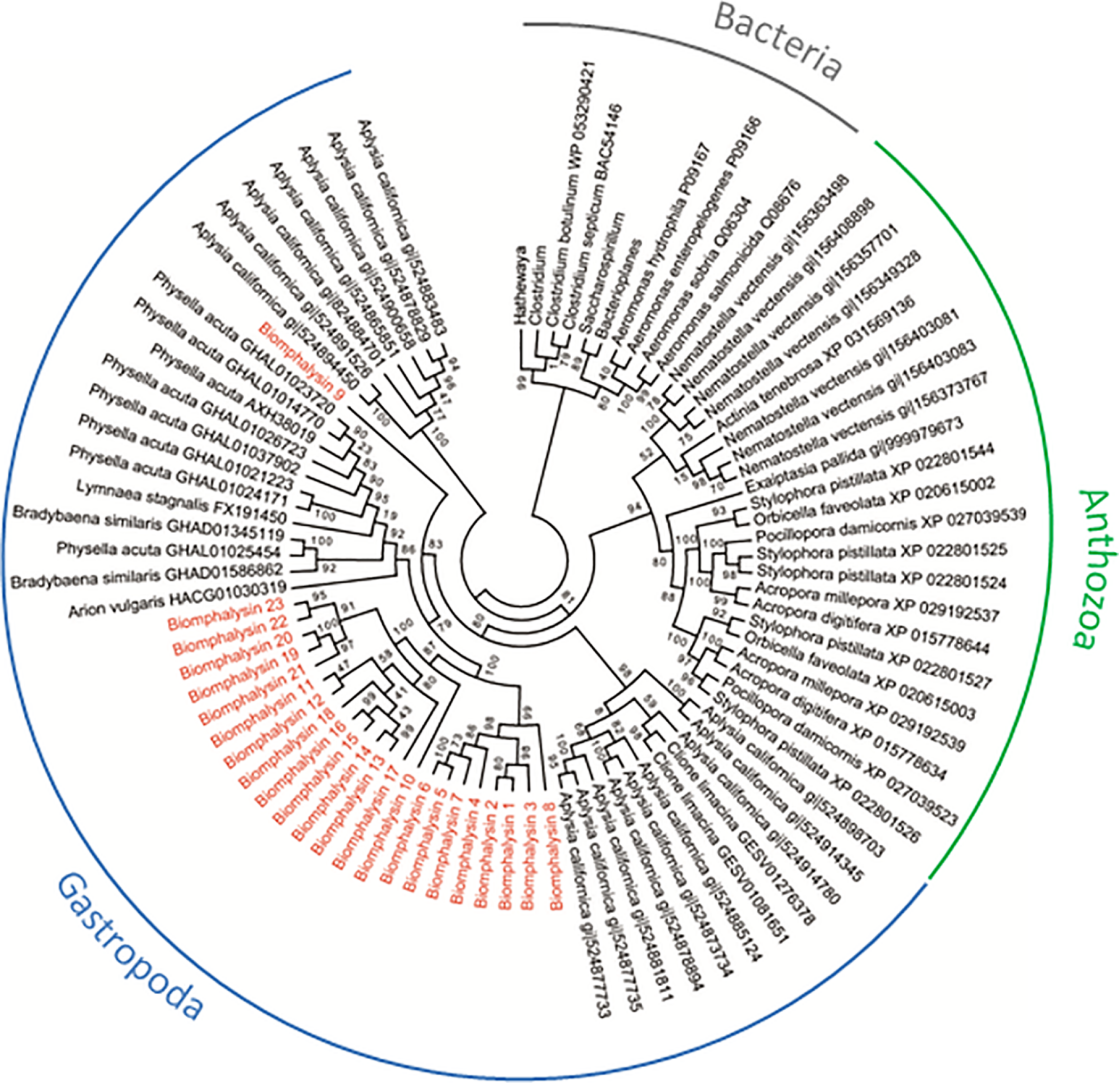
Phylogenetic tree of biomphalysins with other proteins bearing ETX/MTX2 domain. Phylogenetic analysis was conducted from 87 ETX/MTX2 protein domain sequences from biomphalysins and related sequences from other organisms. Protein sequences were aligned using Clustal Omega. Multiple alignment was trimmed using Gblocks, and tree was constructed by Maximum likelihood analysis using PhyML. Values on the branches indicate the robustness of the branches of the tree inferred by ML method. Biomphalysins (red) clustered together with other Gastropoda sequences (blue). Sequences from Anthozoa (green) and Bacteria (gray) formed two other distinct clusters. The accession number of each gene and protein used is listed in **Table S9**.

A second phylogenetic analysis was conducted including the 23 biomphalysin protein sequences together with 52 proteins presenting sequence similarities from other organisms. The aerolysin and MTX2 domains (IPR005830 and IPR004991) were identified in each of the biomphalysin protein sequences by searching against the Conserved Domains Database (http://www.ncbi.nlm.nih.gov/Structure/cdd/wrpsb.cgi) (52). These sequences were used as a query for a BLAST search against the NCBI database to identify similar sequences in other species. A total of 85 sequences were used for the phylogenetic analysis performed as described previously. These included 53 sequences from Gastropoda, 22 from Anthozoa and 10 from bacteria (**Figure 2**). The accession numbers and corresponding names of each sequence used in phylogenetic analysis are indicated in **Table S9** for all phylogenetic trees performed.

### Bioinformatic Analysis

Whole genome and transcriptome screening by use of the Interproscan software were performed with the Interproscan database to find IPR005830 and IPR004991 signature characteristic to aerolysin and aerolysin-like toxins. The genome of *B. glabrata* (preliminary assembly v4.3) and the RNA-seq data from naïve and infected snails were used as data sources (45, 53).

The presence of a signal peptide was determined by a primary structure analysis performed using SignalP 3.0. Protein domain searches were performed using SMART (http://smart.embl-heidelberg.de/) and Motif Scan (http://hits.isb-sib.ch/cgi-bin/PFSCAN) software. α-Helical and β-sheet regions were identified by secondary structure prediction using Jpred3 and HHpred servers (http://toolkit.tuebingen.mpg.de/hhpred). Sequences were searched for putative transmembrane domains (TMDs) using the PRED-TMBB server.

3D Prediction of biomphalysin and their isolated small lobe structures were performed using the I-Tasser V5.1 (https://zhanglab.ccmb.med.umich.edu/I-TASSER/) and TM align https://zhanglab.ccmb.med.umich.edu/TM-align/) servers (54, 55). The 3D structure was obtained by multiple threading using the I-Tasser server, which combines two protein structure prediction methods: threading and *ab initio* prediction (56). Structural similarities between bacterial aerolysin and biomphalysin proteins were determined by calculating a TM-score (**Table S5**). A TM-score greater than 0.5 reveals significant alignment, whereas a TM-score less than 0.17 indicates a random similarity.

Multiple structural alignment were performed using the ConSurfWeb from the predicted structure of different biomphalysins. The conservation scores are projected onto the protein sequence of biomphalysin 1 (**Figure 5**).

### Search for Transposable Elements (TE)

Up to 20Kb region surrounding each biomphalysin (when size and position on the scaffold allowed) was screened for the presence of TEs through a similarity search using BLAST with the *Biomphalaria* specific database of repeat elements as well as using RepeatMasker. We performed a visual inspection for conserved sequences in these 10Kb regions surrounding the biomphalysin genes by producing a dotplot using gepard v. 1.4 (57).

### Transcription Factor Motif Search

Transcription factor motifs were searched using the 2016 version of the JASPAR (58) database of transcription factors (**Table S8**) and the Motif Occurrence Detection Suite MOODS v. 1.9.2 (59) by using the provided script (moods_dna.py) (**Table S7**) and the data was parsed, filtered and summarized by in house developed scripts. The search for conserved domains was carried out by using MEME (60) and searching for motifs between 12 and 50 bases long. Shorter motifs did not produce significant results except for TATA boxes and a poly-C stretch.

### Relative Transcript Abundance in Whole Snails Using RNAseq Experiment

High quality reads were recovered from previously published RNA-seq data (45) and they were aligned on nucleic sequences of the 23 biomphalysins. Briefly, two pools of 20 uninfected snails originating from Brazil (*Bg*BRE) were sampled and RNA was extracted from their whole body using Trizol^®^ Reagent (Sigma Aldrich) prior to Illumina sequencing. Best quality reads (Phred >29) from both libraries (uninfected 1 & uninfected 2) were aligned as previously described (45) on each of the 23 biomphalysins using Bowtie2 (v2.2.4) and samtools (v0.1.18), which were run on a local Galaxy Project server. Raw counts were normalized with upper-quartile division per libraries (61).

### Tissue Representation of Biomphalysin Transcripts

Seven organs (mantel, head-foot, hemocytes, albumen gland, stomach, hepatopancreas and ovotestis) from 5 individual *B. glabrata* snails (*Bg*BRE2) (± 8 mm) were dissected and immediately transferred in liquid nitrogen for mRNA total extraction using Norgen Biotek (Ontario, Canada) RNA extraction kit following the manufacturer’s protocol. DNAse treatment (ThermoFischer, USA) was carried on to eliminate potential traces of contaminant genomic DNA. Total RNA (500 ng) was reverse transcribed (RT) using Maxima H Minus First Strand cDNA synthesis kit (ThermoFischer, USA) following the manufacturer’s protocol. Equimolar random hexamer primer and oligo (dT)_18_ primer were used. Absence of genomic DNA and quality of cDNA synthesis was verified by using myoglobin primers (forward: 5’-GAT GTT CGC CAA TGT TCC C-3’ and reverse: 5’-AGC GAT CAA GTT TCC CCA G-3’) targeting intron/exon junctions. All primer couples targeting biomphalysin genes were designed with perlprimer software (**Table S2**). Specificity of amplification was validated by Sanger sequencing of amplicons. For tissue-specific expression, PCR amplifications were performed on the 7 organs in biological quintuplicate on cDNAs (diluted 5-fold with nuclease-free water). PCR reactions were performed with GoTaq^®^ G2 Hot Start Polymerase kit (Promega, Madison, USA) and performed on a thermocycler (Eppendorf, Hamburg, Germany). PCR protocol was as follows: 4 min at 95°C for initial polymerase activation then 35 cycles constituted of 20s denaturation at 95°C, 30s annealing at 48°C and 30s extension at 72°C with 5 min at 72°C of final extension. PCR products were separated by electrophoresis on 1.5% agarose gels and sequenced.

### Immune Challenge Experiments

Snail (*Bg*BRE2) exposed to Gram-positive, Gram-negative bacteria or yeast were conducted according to previously described procedures (62). Briefly, snails were bathed with 10^8^/mL of each microorganisms for 1 h. Then, snails were washed extensively. For *S. mansoni* infection, each snail was exposed for 6 h to 10 miracidia from Guadeloupe (*Sm*GH2) in 5 mL of pond water. In this snail/parasite interaction, *Sm*GH2 is totally incompatible with *Bg*BRE2. For each immune challenge, 12 replicates of a pool of three snails were performed at 6, 12, 24 and 48 h after exposition. Non-exposed snails (8 replicates of a pool of three snails) were used as control for the evaluation of the basal expression level of tested biomphalysins.

### Quantitative Real Time PCR Assay for Biomphalysins Expression Analysis Upon Immune Challenge

RNA extractions were performed according to the Trizol™ Reagent procedure (ThermoFisher Scientific, Paris, France). DNase treatment and reverse transcription of RNA (4µg) into cDNA were performed with Maxima H minus first strand cDNA synthesis kit (ThermoFisher Scientific, Waltham, MA, USA) using random hexamer according to the manufacturer’s instructions. Real-time RT-PCR analyses were performed using the LightCycler 480 System (Roche) performed in the same way as described in (63). Only primer couples (**Table S2**) with amplification efficiency of 2 were retained. The cycling program is as follows: denaturation step at 95°C for 2 min, 40 cycles of amplification (denaturation at 95°C for 10 s, annealing at 60°C for 15 s, and elongation at 72°C for 22 s). QPCR was ended by a melting curve step from 65 to 97°C with a heating rate of 0.11°C/s and continuous fluorescence measurement. For each reaction, the cycle threshold (*C*_t_) was determined using the second derivative method of the LightCycler 480 Software release 1.5 (Roche). A mean value of *C*_t_ was calculated. The relative expression of biomphalysins was normalized to the housekeeping gene S19 ribosomal protein gene used as reference gene. Then, normalized expression of biomphalysins was compared with non-exposed snails (used as control) with the ΔΔCt method. The normality of the dataset was tested using Shapiro Wilk test, and significant differences were analyzed by pairwise Mann Whitney U test. Differences were considered significant and robust when p<0.005.

### Codon Usage and GC Content

Codon usage were compared between sequences from the 23 biomphalysins, 13 aerolysins/Pertussis Toxin for bacteria and 25 randomly selected *B. glabrata* predicted transcript (1600-1800 pb) (**Table S10**). The size of *B. glabrata* transcripts was chosen to be in the range of biomphalysin genes (1600-1800 pb) to avoid biases due to differences in gene length. Nucleotide sequences were compared using codon usage tools of CAIcal server (http://genomes.urv.es/CAIcal/) (64). Mean codon usage was compared for each triplet between biomphalysin transcripts, *Biomphalaria* transcripts and bacteria transcripts by PCA (Principal Component Analysis) using ClustVis web tool (http://biit.cs.ut.ee/clustvis/) (65). Local transcriptome of *B. glabrata* (45), the 23 nucleic sequences of biomphalysins and 13 aerolysin-like sequences were used to calculate the GC content using geecee tools (v5.0.0) on local Galaxy server.

### Biomphalysins Identification in an Interactome Approach Between Pathogens and Snail Hemolymph

Interactome experiments were conducted to determine which snail plasma proteins are capable of binding to the surface of pathogens. Experimental procedure was previously described (66). Briefly, hemolymph was collected from the head–foot region of unexposed snails. Hemolymph was centrifugated to keep only the free-cell compartment which was incubated during 20 min at 26°C with living organisms such as *E. coli, M. luteus, S. cerevisiae, S. mansoni and E. caproni*. Then, organisms were washed twice with CBSS (Chernin’s balanced salt solution) and resuspended in a lysate buffer (urea, 7 M; thiourea, 2 M; Tris, 30 mM; CHAPS, 4%; pH 8.5) to extract proteins from organisms but also snail plasma proteins associated with their membrane. Extracted proteins were subjected to 2D electrophoresis and silver staining. Extracted proteins from living organisms exposed to CBSS were used as control. Five independent experiments were performed. Then, a comparative analysis between 2D gels from non-exposed and exposed organisms to snail cell-free hemolymph was performed. Only additional spots present in all the three replicates of “organisms exposed to cell-free hemolymph” samples and absent from all the profiles of pathogens alone were collected and submitted to mass spectrometry analysis for identification. To identify biomphalysin proteins present in these additional spots, the obtained peptides by mass spectrometry analysis (nano LCMS/MS, nano-LC1200 system coupled to a Q-TOF 6550 mass spectrometer) were matched to all translated transcripts encoding the different biomphalysin proteins.

## Results

### From One Biomphalysin to a Family of 23 Genes

To identify all of the gene encoding analogues of aerolysin toxins *in B. glabrata*, we conducted an exhaustive sequence search of the genome and transcriptomes of *B. glabrata*. Using the gene sequence from the previously identified biomphalysin 1 (42), only a single partial sequence of 395 bases (Locus_25218_Transcript_19/20) was identified from a local BLAST search of RNA-seq data from uninfected snails (53). However, a tBLASTn search using the same query and database allowed 10 additional hits to be found, with potential homologies to biomphalysin 1 with an E-value score ranging from 1e-79 to 5e-38. As most of these hits harbored an aerolysin or epsilon toxin signature in their sequence (InterPro accession: IPR005830 and IPR004991, respectively), InterProScan and sequence similarity searches were conducted to investigate the presence of additional aerolysin-like genes in the genome of *B. glabrata* (preliminary assembly v4.3) (46) and the RNA-seq data from *S. mansoni* infected snails (45). A total of 23 unique loci were identified on 20 different genomic scaffolds (**Table S1**), including the biomphalysin gene named biomphalysin 1 as well as others, named from biomphalysin 2 to 23. Interestingly, all of the biomphalysin genes identified were devoid of introns, which represents an evolutionary conserved characteristic of this gene family in *B. glabrata*.

Several biomphalysins were found in the same scaffold. Biomphalysins 1 & 2, 11 & 22 and 20 & 21 were located in tandem in large scaffolds, scaffold 10 (1 Mb), 2594 (72 kb) and 2201 (88 kb), respectively. This suggests that these gene pairs could result from duplication events. Interestingly, although present on different scaffolds, biomphalysins 5 and 6 exhibited a high sequence homology of the two gene parts located in the 5’ and 3’ regions of the genes, as evidenced by comparative sequence dot blot analyses performed with Gepard (**Figure S1**). A similar observation was made for biomphalysins 11 and 22 in the 5’ part of the sequence. No other highly conserved regions were detected by Gepard analysis including signature of transposable elements in the 20 Kb vicinity of biomphalysin genes, which was also investigated by using RepeatMasker software and by performing BLAST searches against the repeat database (Dfam and Repbase).

### Biomphalysins Represent a Highly Diverse Family in *B. glabrata*

To rule out potential errors in genome sequencing and assembly, each biomphalysin gene was amplified and sequenced by Sanger sequencing. PCRs were performed on snail genomic DNA using specific primer pairs to amplify the predicted coding DNA sequence of each biomphalysin gene (**Table S2**). The putative translated regions were 1725 bp long on average (1698 -1758 bp). The newly identified biomphalysins exhibited a 46.8 to 94.7% and 33.1 to 95.1% identity as compared to biomphalysin 1 based on the nucleic and deduced amino acid sequences, respectively (**Tables S3** and **S4**). Moreover, protein sequence similarities to aerolysin toxin (Accession number: AAA21938) ranged from 23.6 to 30.2% (**Table S4**). The lowest identity was found between biomphalysins 9 and 23 (28.7%), while the highest was observed for biomphalysins 1 and 2 (95.1%). Their level of pairwise similarity was also very dissimilar, ranging from 43.1% between biomphalysins 9 and 23 to 97.7% between biomphalysins 1 and 2 (**Table S4**).

The deduced amino-acid sequences of the 23 biomphalysins were compared by performing a phylogenetic analysis. At least three clusters of biomphalysins could be observed and they were supported by high bootstrap values (**Figure 1**). The first cluster included biomphalysins 1 to 8, the second biomphalysins 11 to 18 and the third included biomphalysins 19 to 23. Biomphalysins 9 and 10 were isolated from the others and biomphalysin 9 was the closest to the aerolysin sequence of *Aeromonas hydrophila* used as an outgroup (**Figure 1**).

### Biomphalysins Exhibit Shared Domains and Cluster-Specific Features

Biomphalysin proteins are composed of an average of 575 residues (from 565 to 585 amino acids) and all harbored a predicted N-terminal signal peptide for secretion composed of 17-23 amino acids that adopted an alpha-helical conformation (**Figure S2**). A structural feature shared by all aerolysin-like toxins is the presence of many β-sheet elements that represent approximately 40% of the total sequence. Most of them are present in the common core, also called aerolysin domain, consisting of five β-strands with an insertion loop corresponding to the pore-forming transmembrane domain (TMD) (**Figure S2**). This TMD is found in all biomphalysins and is shared by members of the β-PFTs as it plays a crucial role in the formation of amphipathic barrels by oligomerization for membrane insertion (67, 68). This predicted membrane spanning domain flanked by two polar residue regions is found in all biomphalysin sequences (**Figure S3A**). Moreover, an alternating pattern of hydrophobic and hydrophilic residues located in this beta-hairpin loop is conserved in all biomphalysins, highlighting their ability to perforate the lipid layer of targeted organisms (**Figures S3B, C**) (69). Interestingly, the amino acids involved in oligomerization and cytolytic activity were highly conserved in all biomphalysin genes (**Figure S2**). By analogy with site-directed mutagenesis performed on aerolysin proteins, amino acids Asp_235_ and Cys_255_ of biomphalysin 1 could fulfill a crucial role in the oligomeric assembly into a ring-like structure (70, 71). While Tryp_466_ and Tryp_468_ mutations may modify the ability of the aerolysin toxin to oligomerize (72) Tryp_420_ and His_428_ could be involved in binding to membrane target moieties (72, 73). The C terminal part of the protein tends to be divergent between some biomphalysins and could be associated to a differential lytic activation (74).

### Species-Specific Diversity of Aerolysin-Like Proteins Among Invertebrates

To determine whether such an expansion and/or diversification of the aerolysin-like genes occurred in other invertebrates, we also searched in several Sequence Databases of NCBI (Nucleotide, Protein, EST). Next, a phylogenetic analysis was performed using sequences of the aerolysin domain of aerolysin-like proteins from two bacteria genera *Firmicutes and Gammaproteobacteria* and different invertebrates, including the Gastropoda *B. glabrata*, *Aplysia californica*, *Physella acuta*, *Bradybaena similaris*, *Lymnaea stagnalis* and *Arion vulgaris*, and the Anthozoa *Nematostella vectensis*, *Exaiptasia pallida*, *Pocillopora damicornis*, *Stylophora pistillata*, *Acropora millepora*, *Acropora digitifera*, *Orbicella faveolata* and *Actina tenebrosa* (**Figure 2**). Bacterial toxins all clustered together, with two different sub-clusters corresponding to the two bacterial genera used, and they were clearly separated from invertebrate aerolysin-like sequences (**Figure 2**).

For invertebrate sequences, the phylogenetic analysis segregates aerolysin-like proteins from Gastropoda and Anthozoa. Interestingly, the phylogenetic closeness of species did induce a higher phylogenetic similarity in the sequences of aerolysin-like proteins. Main branches present relatively good support values (ranging from 79 to 100%) (**Figure 2**). Aerolysin-like proteins from the Gastropoda *A. californica* were more similar to those from *C. Limacina* as they both belonged to Opistobranchs class. At the opposite, the five others gastropod species which belong to Panpulmonata clade present higher phylogenetic similarities of their aerolysin-like proteins. On the same way in Anthozoa class, aerolysin-like sequences from hard corals species (Scleractinia) and from soft coral ones (Actinaria) formed two different clusters. Aerolysin-like proteins cluster according to the taxonomic classification of the organism from which they have been identified. This suggests that the multiple toxins of each species could result from a species-specific diversification. However in *B. glabrata*, biomphalysin 9 was not clustered with the 22 other biomphalysins and instead, positioned at a basal node of all the other aerolysin-related proteins from panpulmonata species, suggesting a markedly different evolutionary process between the biomphalysin genes. Also, aerolysin-like proteins from *A. californica* are separated into two different clusters for which one is more basal than aerolysin-like proteins from all other invertebrate species used in this study.

### Biomphalysins Result From an Old Domestication of Aerolysin-Like Genes

**Figure 3:**
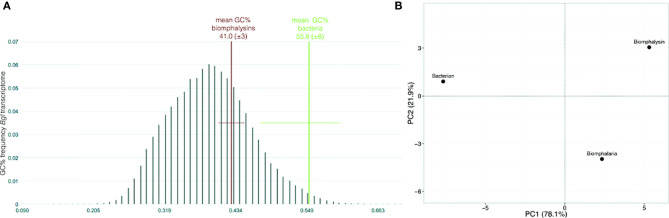
Nucleotide support for domestication. **(A)** Box-plot representation of GC content and **(B)** principal component analysis on codon usage comparing nucleotide sequences of biomphalysins (N = 23) with other *Biomphalaria glabrata* sequences (± 1700 bp; N = 25) and bacterial toxin sequences (N = 13). Distribution of GC% was indicated for *Biomphalaria glabrata* transcripts while only the mean and standard deviation were indicated for biomphalysin and bacterial toxin sequences.

GC content and the codon usage are genus-specific signatures and common features used to detect recent horizontal transfer events by comparing the sequences of the gene supposedly acquired by HGT (here, biomphalysins) (42) and gene sequences from the recipient species (here, other genes from *B. glabrata*). The GC content of biomphalysin genes (41.0%) was similar to *B. glabrata* transcripts but was significantly lower than the GC% of bacterial toxin nucleic sequences (55.8%, Fisher’s exact test, p-value = 0.0232) (**Figure 3A**). Concerning the codon composition, most of the variance (78.1%) was explained by the first axis of the principal component analysis (PCA), which opposed bacterial sequences to snail ones, while the second axis which explained 21.9% of variance, separated the biomphalysins from other genes of *B. glabrata* (**Figure 3B**). GC% and codon usage of biomphalysin and bacterial toxin genes have distinct signatures and their analysis indicates that biomphalysins are more closely related to endogenous *Biomphalaria* transcripts than to bacterial toxin genes, which suggests an old domestication of biomphalysin genes by the snail.

### Highly Conserved Three-Dimensional Structures Despite a High Variation in Sequence Conservation

**Figure 4:**
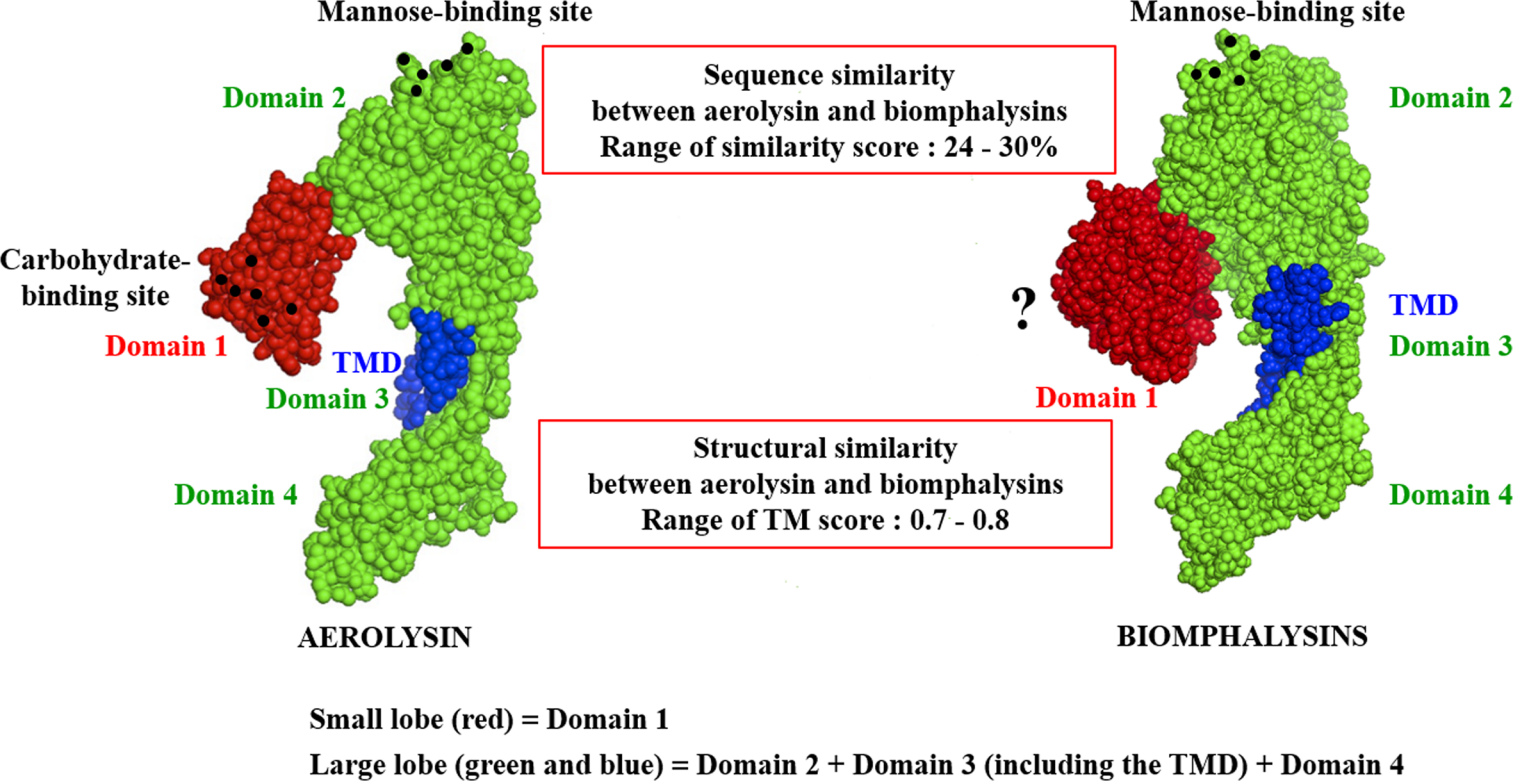
Structural schematic representation of biomphalysins. Images of 3D structure of aerolysin (PDB accession code: 1PRE) and predicted biomphalysin 1 were generated using PyMOL in sphere style. The small domain of both proteins is colored in red, the large domain in green and the TMD in blue. Amino acid residues involved in glycan recognition are symbolized by black spheres. “?” means that no motif has been detected using motif prediction software such as the SMART and MotifScan programs.

**Figure 5:**
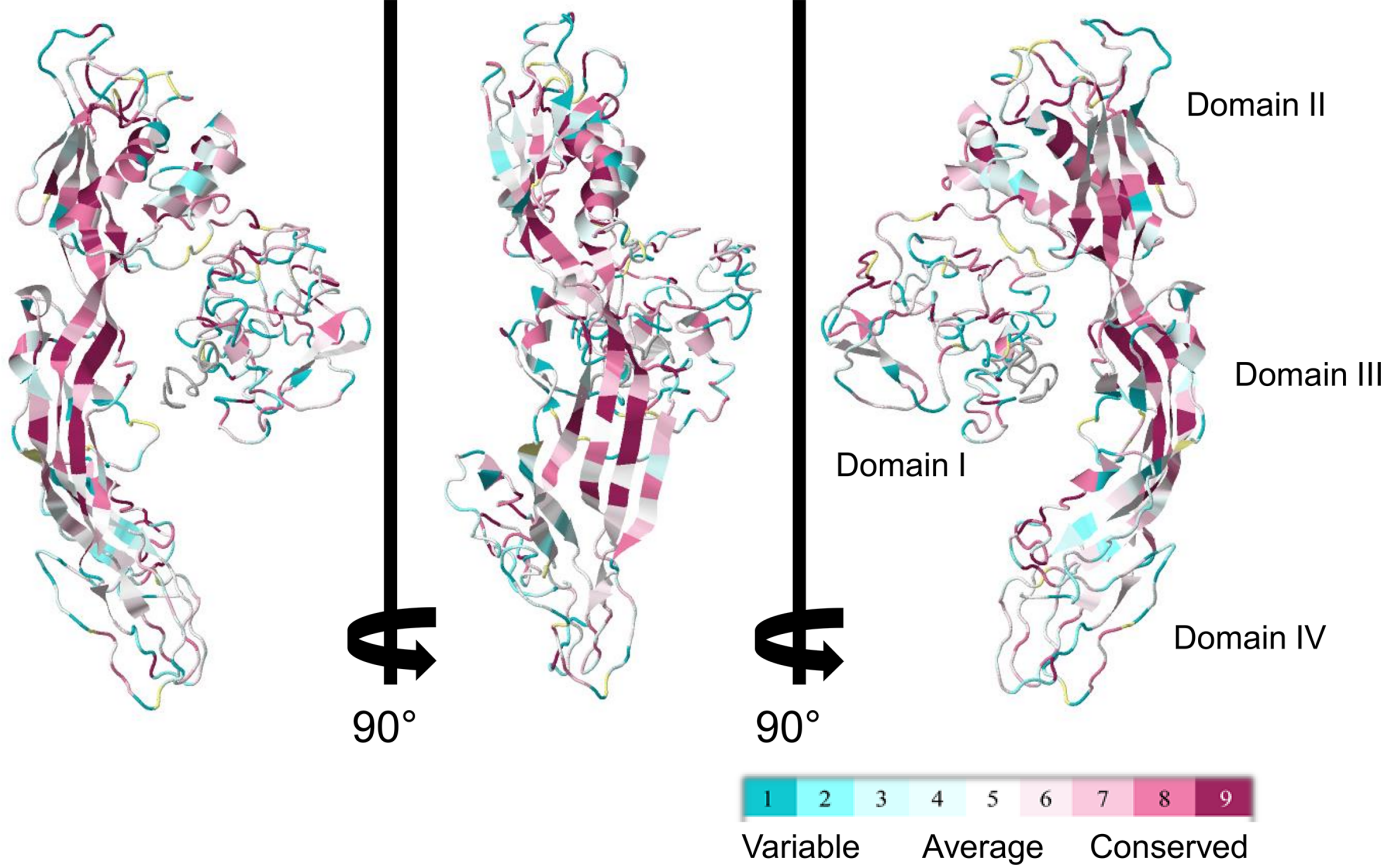
ConSurf sequence conservation score of biomphalysin proteins. ConSurf image was generated using alignment of all 23 biomphalysin proteins and by plotting alignment scores on the predicted structure of biomphalysin 1. Variable residues are shown in cyan while the most conserved ones are in dark red. Images are rotated 90° around the y axis.

Despite the apparent divergence and the low sequence homology between the 23 biomphalysins, their structures exhibit high levels of similarity (**Figure 4**). We previously reported that the anti-parasitic biomphalysin 1 displayed the same structural features as the bacterial aerolysin toxin (42). A structural modeling for each biomphalysin protein was performed using the best alignment template against the Protein Data Bank (PDB) database (http://rcsb.org/) using the I-Tasser server (54, 75). Structural similarities are indicated by a TM score ranging from 0 (totally dissimilar proteins) to 1 (exactly similar structure). All biomphalysins exhibited a structure close to the aerolysin with a TM score ranging from 0.64 to 0.80 (**Table S5**). The analysis allowed for the prediction of two distinct lobes like for the aerolysin toxin from *Aeromonas hydrophila*: a small lobe named Domain I and a large lobe constituted of three structural domains (Domain II, III and IV) (**Figures S2**, **4**). Despite differences in the primary structure, highly similar conformations were observed between the different members, with an average pairwise TM score of 0.77 (**Table S5**). An analysis of the conservation of residue positions among the biomphalysin family was performed with the Consurf software (**Figure 5**) (76). Conservation scores mapped onto the 3D structure of biomphalysin 1 revealed that most conserved residues are located in Domain II, which is involved in oligomerization (70) and moiety binding in aerolysin (77, 78), and in Domain III, which is necessary for the stabilization of the channel (79) and for toxin insertion into lipid bilayers *via* the transmembrane α-hairpin loop (67). Conserved residues are strategically located to maintain the highly organized typical topological arrangement of a β-PFT. Indeed, the most conserved residues shape the two α-helices and the different β-strands from Domains II and III (**Figures S2**, **5**). The structure of this large lobe appears to be conserved despite a divergent sequence with an average TM score of 0.93 ± 0.02 (**Table S6**). Interestingly, many variable amino acids were found in loop regions and particularly in the loop from TMD (**Figure 5**). Nevertheless, the stretches of alternating hydrophobic and hydrophilic residues, which are key elements for the transmembrane feature, are also conserved (**Figure S3B**).

Considering that structural information revealed the presence of variable loops mostly located in Domain II, which could contribute to the specificity of interaction with its target, protein-ligand binding sites were predicted based on biomphalysin three-dimensional structures. In addition, a mannose-6-phosphate binding domain was identified in Domain II of all biomphalysins (**Figure S4**). This carbohydrate binding pocket is located in the vicinity of the two α-helices, a well-characterized site in aerolysin toxin involved in the binding of the mannose residue from glycosyl phosphatidylinositol (GPI)-anchored receptors (78). It contains several key residues for its binding activity, such as arginine, glycine and tryptophan (**Figures S2**, **S4**) (68, 80, 81).

### Small Lobe Shares High Structure Similarity With Immune Recognition Domains

The small lobe (Domain I) of the aerolysin toxin presents a binding site similar to the carbohydrate-binding domain of C-type lectins, also called Aerolysin Pertussis Toxin (APT) domain (IPR005138) (26). For all biomphalysin proteins, no lectin motif was found in the amino acid sequence. As most residues were variables in the small lobe of biomphalysin proteins, a similarity structural search was performed using the I-TASSER software against proteins whose tridimensional structures were resolved by crystallography (54). TM scores for the isolated small lobe ranged from 0.45 to 0.87 and were therefore much lower than those of the large lobe (0.89 to 0.97) (**Table S6**). No significant predictions were obtained for four of the small lobes as their similarity score was below the threshold of 0.5, considered as the limit of prediction significance (54). The analysis of the small lobes of the nineteen other biomphalysins gave relevant structural alignments to five different crystallized proteins or domains of crystallized proteins (**Table 1**).

**Table 1:** Best predicted hit for the structure of the small lobes of each biomphalysin.

	Top hit of I-TASSER structure predictiongenerated from the small biomphalysin lobe
Biomphalysin 1	TM-score < 0.5
Biomphalysin 2	Complement factor B
Biomphalysin 3	Virus capsid
Biomphalysin 4	Complement factor B
Biomphalysin 5	Apo form cyclase
Biomphalysin 6	Complement C3b in complex with Factors B and D
Biomphalysin 7	TM-score < 0.5
Biomphalysin 8	Virus capsid
Biomphalysin 9	Complement factor B
Biomphalysin 10	TM-score < 0.5
Biomphalysin 11	Complement factor B
Biomphalysin 12	Complement C3b in complex with Factors B and D
Biomphalysin 13	Complement factor B
Biomphalysin 14	Complement factor B
Biomphalysin 15	TM-score < 0.5
Biomphalysin 16	Complement factor B
Biomphalysin 17	Complement factor B
Biomphalysin 18	Complement factor B
Biomphalysin 19	Complement factor B
Biomphalysin 20	PKh Kinase Domain
Biomphalysin 21	Complement factor B
Biomphalysin 22	Complement C3b in complex with Factors B and D
Biomphalysin 23	TM-score < 0.5

Prediction structure was performed using the I-Tasser server and the structure alignment was realized between the first I-Tasser prediction and the most similar structure templates in PDB.

**Figure 6:**
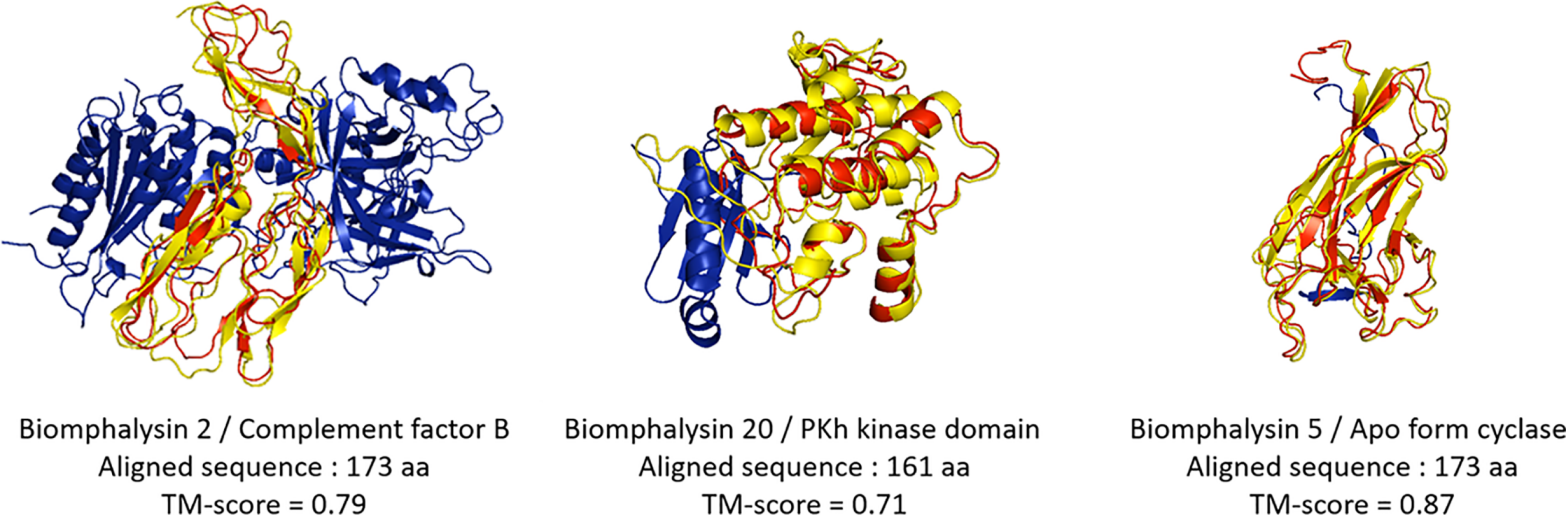
Structural prediction of the biomphalysin small lobes. Three-dimensional structure prediction of small lobe of biomphalysin proteins 2, 5 and 20 was performed using I-Tasser server. A C-score was determined for each prediction (see **Table S7**). The small lobe of biomphalysin proteins 2, 5 and 20 were investigated against RCSB Protein Data Bank (PDB). A TM-score was calculated between the small lobes and the best hit found by I-Tasser server. A score greater than 0.5 reveals significant alignment. 3D superposition of each small lobe of biomphalysin 2, 5 and 20 and the respective best hit were performed by TM align and visualized with PyMOL. The best structural alignment was reported between the predicted isolated structure of the small lobe in red and the significant template hit identified in yellow.

Despite an overall similar architecture, the small lobe of the different biomphalysins was predicted to fold into different states (**Figure 6**). For example, the small lobes of the biomphalysin 3 and 20 were respectively similar to a capsid protein and a kinase domain of the PDK1 (phosphoinositide-dependent protein kinase 1), that shaped a protein substrate docking site (82, 83). Eleven of the small lobes matched with a high TM score (higher than 0.6) to members of the complement system, especially complement factor B, which is part of the innate immune system that enhances the immune response against pathogens through their interacting features (84). Interestingly, the best hit of structural prediction for four of the remaining biomphalysins corresponded to a crystal of a protein complex composed of the complement fragment C3b, Factors B and D. In this complex, the small lobes of biomphalysins 6, 12, 15 and 22 are structured in the same way as the domain Ba of Factor B, characterized to interact with the terminal end of C3b, mainly with the MG7 (MacroGlobulin) and the CUB (complement C1r/C1s, Uegf, Bmp1) domains (85). This indicates that the structural diversification of the small lobe might have led to a functional diversification of biomphalysins. This small lobe presents the highest variability of folding units that are independent from the large lobe, leading to a potential broad spectrum of targeted organisms. This unit module could be considered as a diversified moiety-binding domain associated with the aerolysin domain that is involved in the lytic function.

### Each Biomphalysin Is Expressed and Exhibits a Specific Tissue-Expression Pattern

**Figure 7:**
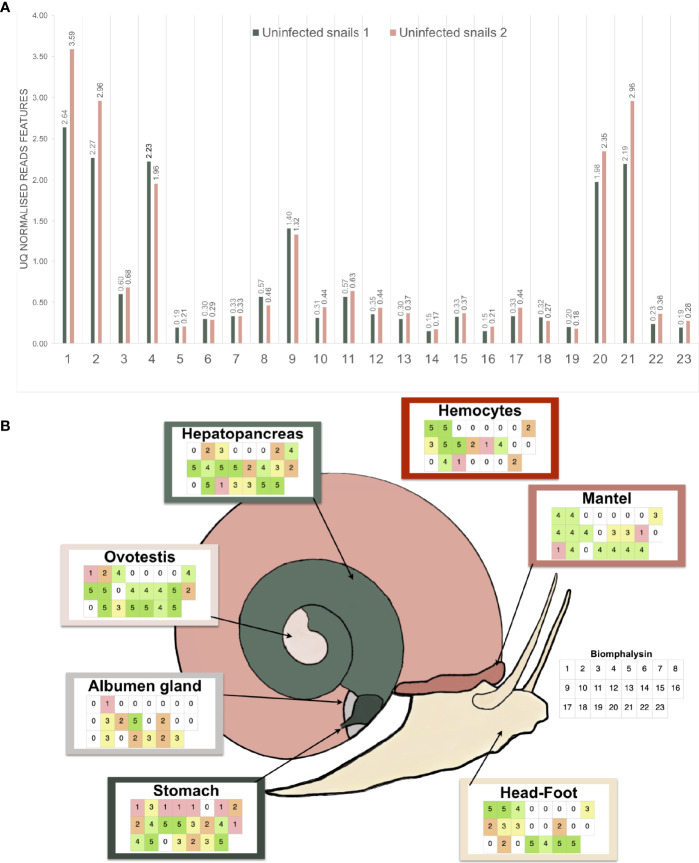
Expression analysis of the biomphalysin family. **(A)** Constitutive expression of biomphalysins members in whole snails rnaseq dataset (Pinaud et al., 2016). Transcripts abundance for all 23 biomphalysins retrieved from RNAseq experiment performed on two pools of 20 uninfected snails. Libraries from uninfected snails 1 and 2 are highlighted in light-green and pink, respectively. Counts were normalized by upper-quartile (UQ) division for each library. **(B)** Patterns* *of expression of biomphalysins in different snail tissues. Each table represents the presence of the 23 biomphalysin transcripts in each tissue by RT-PCR, from biomphalysin 1 in the upper left case to biomphalysin 23 in the lower right case of each table. A code color was used and corresponds to the number of snails for which the tested biomphalysin was detected in a given tissue. As example, an uncolored case indicates that the biomphalysin studied was not expressed in any snail.

High quality reads from a previous RNA-seq experiment that included two pools of 20 uninfected snails were used (45). They were aligned with high-stringency to the 23 biomphalysin genes using Bowtie2, counted and normalized by upper-quartile for each library. Every biomphalysin was detected in uninfected snails with different levels of expression (**Figure 7A**). Biomphalysins 1, 2, 4, 20 and 21 were the most represented whereas biomphalysins 5, 14 and 16 were the least expressed. Transcription of all biomphalysins highlights that none of them are pseudogenes.

To further explore the patterns of expression of the biomphalysin genes, we analyzed their transcript representation by RT-PCR in seven different tissues (stomach, albumen gland, ovotestis, hepatopancreas, hemocytes, mantel, head-foot). This revealed a mosaic pattern of expression of these genes in naïve snails at tissue and individual levels (**Figure 7B**). While some biomphalysins, such as biomphalysins 2, 10, 14, 18 and 23, were ubiquitously expressed in all tested tissues, others were specifically expressed in some tissues, such as biomphalysin 7, that was restricted to the hepatopancreas and stomach or the biomphalysin 17, which was only located in the mantel and stomach (**Figure 7B**). Moreover, a high variation in the number of biomphalysins expressed could be observed between the tissues. The highest number of biomphalysins expressed was observed in the stomach, which contained transcripts for 22 out of the 23 biomphalysins, while albumen gland and hemocytes solely displayed 11 and 12 biomphalysins expressed, respectively. Such high variability in tissue expression suggests that the different biomphalysins might have different functions. Many transcriptional factor binding sites were identified in the promoter regions of the biomphalysins but none were found conserved for all the 23 biomphalysins (**Tables S7**, **S8**). The diversity of the *cis* regulatory elements found in the promoter regions suggests that each biomphalysin gene could be regulated spatially and temporally by specific transcription factors, supporting the hypothesis of a functional divergence in this family (**Table S8**). Consequently, we suggest that each biomphalysin gene could be associated with a functional diversification and sub-functionalization of the snail’s innate immune system to mount an efficient response to different pathogens or to regulate host–commensal bacteria communities or may even be involved in non-immune functions, such as cellular turn-over or nutrition.

### Effect of Immune Challenge on Biomphalysin Gene Expression

**Figure 8:**
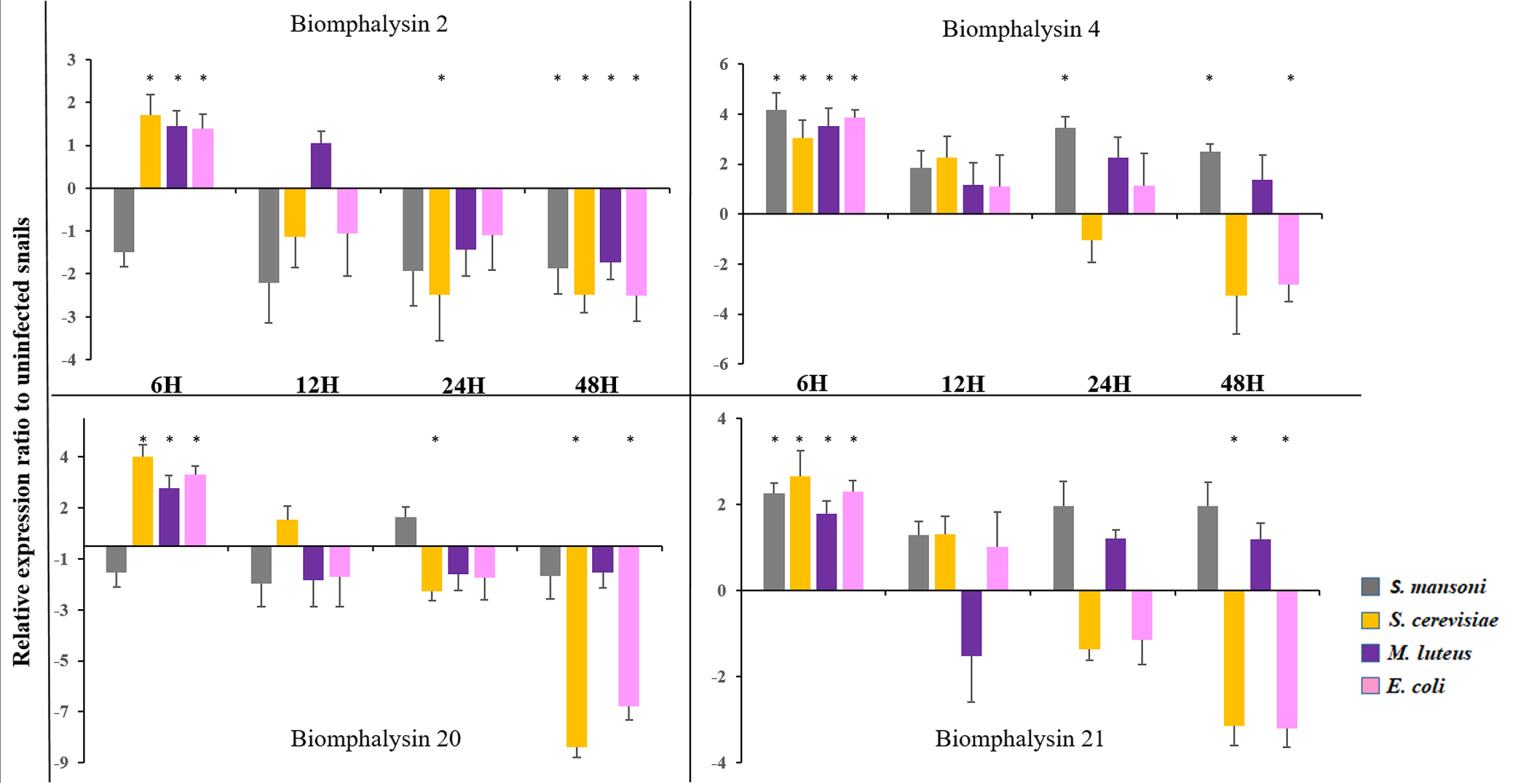
Expression of Biomphalysin 2, 4, 20 and 21 Expression of biomphalysins was monitored 6, 12, 24 and 48 h after immune challenge with *Schistosoma mansoni* (gray), *Saccharomyces cerevisiae* (orange), *Micrococcus luteus* (purple) and *Escherichia coli* (pink). Expression normalization was done using the S19 housekeeping gene for each experimental point. Data are represented as mean 2^- ΔΔCt^ in challenged snails relative to unexposed snails. Values of 2^- ΔΔCt^ less than 1 were transformed for a better visualization as follow: -1/(2^- ΔΔCt^). * indicates a significant over- or under-expression in challenged condition compared to control (Mann-Whitney U test, *p* < 0.005).

To explore the role of biomphalysins in immunity against different pathogens, snails were exposed to either bacteria, yeasts or Schistosoma parasites to investigate the effects of immune challenges on the expression of different members of the biomphalysin family. We focused on the most expressed biomphalysins in uninfected snails (**Figure 7A**) and measured their relative expression ratio by RT-qPCR in response to an exposure of potential pathogens (**Figure 8**). Expressions of biomphalysins 2, 4, 20 and 21 are significantly up-regulated at 6h post-microbial exposure. Among the biomphalysins monitored, microorganism-induced expression of biomphalysin 4 is the highest from 3 to 4 fold (p< 0.005). When compared to the control, after 6 hours of exposure, their expression tends to decrease with a significant reduction at 48h post-exposure with *E. coli* and *S. cerevisiae* challenges for biomphalysins 2, 20 and 21 (from 2.5 to 7.8 fold). Interestingly, the expression of biomphalysin 4 and 21 are induced significantly 6h after *S. mansoni* exposure, 2.3- and 4.2-fold respectively. Their level remains elevated compared to the control for up to 48h, with a significance for biomphalysin 4 overexpression (3.3- to 2.6- fold at 24 and 48h, respectively). Unlike to biomphalysin 1 expression which is not modulated by Schistosoma exposure (42), biomphalysins 4 and 21 may play a role in the response to parasite infection by forming a homo- or heteromultimeric pore complex to sustain a lytic response. Taken together, our findings suggest that biomphalysins repertoire may be crucial to mount an efficient and specific immune response against various pathogens.

### Different Biomphalysins Are Involved in the Recognition of a Large Diversity of Pathogens

As changes in gene expression do not always correlate with changes at the protein level, investigations of the interaction of biomphalysins with pathogen proteins during infection are required. A recent interactome approach was performed by confronting soluble proteins from the snail cell-free hemolymph with outer membrane proteins of living pathogens (66). This approach allowed for the identification of biomphalysin as a pathogen-interacting protein (66). Biomphalysins interact *in vivo* with gram-negative (*Escherichia coli*) and gram-positive bacteria (*Micrococcus luteus*), yeast (*Saccharomyces cerevisiae*) and two metazoan parasites, *Echinostoma caproni* and *S. mansoni*) (66). Using peptide MS/MS spectra recovered from the 2D-gels spots identified as biomphalysin, we sought to identify the biomphalysins that were associated with the different pathogen tested, by mapping them onto the deduced amino acid sequences of the 23 biomphalysins (**Table 2**). Some peptides were identified in 4 out of 5 interactions, and others were only present in a single interaction. Next, we calculated the number of biomphalysins potentially involved in the interaction with each pathogen tested. *E. coli* and *E. caproni* were recognized by at least one biomphalysin, and a minimum of two biomphalysins were required to sense the presence of *M. luteus*, *S. cerevisiae* and *S. mansoni* (**Table 2**). This is consistent with the fact that three different spots were identified in the gels for these three pathogens; thus, in contrast with what we initially thought, this suggests that two different biomphalysin members - and not different isoforms of the same biomphalysin - are involved in binding to different pathogens (66).

**Table 2:** Biomphalysin identification revealed by an interactome approach between pathogens and cell-free hemolymph.

Peptide List	*M. luteus*	*E. coli*	*S. cerevisiae*	*S. mansoni*	*E. caproni*
LTDETQYQFTLTGK	B2, B3, B4, B5, B6, B7		B2, B3, B4, B5, B6, B7	B2, B3, B4, B5, B6, B7	B2, B3, B4, B5, B6, B7
FGDSSVPFYK	B1, B2		B1, B2	B1, B2	B1, B2
ADGDDLYFLK	B1, B2, B3, B4, B7		B1, B2, B3, B4, B7		B1, B2, B3, B4, B7
ADGDDLYFLKK	B1, B2, B3, B4, B7		B1, B2, B3, B4, B7		B1, B2, B3, B4, B7
QSSITLGPMEAAK	B1, B3	B1,B3			
ASSPVTESIER	B1, B3, B5, B6			B1, B3, B5, B6	
LEKVEGTSVNVK			B1, B2		B1, B2
TTVPYTAIITTK			B1, B2, B3		B1, B2, B3
SVIEDLQAESVDSGVLYNR			B1, B2		B1, B2
SVIEDLQAESVDSG			B1, B2, B3		
CCTPAAKPLEMDEK			B1, B2, B5		
SISETQGFTK			B1		
FEYSTSTTNSK			B1		
TRFEYSTSTTNSK			B1		
YQVIMSK		B1, B3			
SISQTTGFTK					B2, B7
**# spots identified as biomphalysin**	3	1	3	3	3
**Min. # of biomphalysins involved**	2	1	2	2	1

## Discussion

In natural environments, *Biomphalaria glabrata* snails face complex communities of pathogens. It has been extensively demonstrated that *B. glabrata* are confronted with metazoan parasites (nematodes or trematodes) and play the role of the intermediate vector in the human *Schistosoma* life cycle (86–89). Albeit rare, crustacean infection has been experimentally demonstrated too (90). Despite being pulmonated, *Biomphalaria* snails are dependent on wetland ecosystems and must also be confronted with fungal (91) or microbial infections (92). Finally, it has also been demonstrated that *B. glabrata* may be associated with complex endosymbiotic bacterial microbiota (93–97) or numerous endosymbiotic viruses (98). Since Biomphalaria is one of the main intermediate hosts of *Schistosoma*, significant efforts have been made to decipher the interaction between the host’s defense mechanisms and the parasite’s infective strategies. Genome sequencing and annotation have made it possible to predict which genes are potentially responsible for the encoding factors involved in snail immune defense (46, 99). Based on comparative approaches between susceptible and resistant snail strains, several candidate genes were proposed as those involved in parasite recognition or elimination (100–104). In addition, small RNA-mediated gene silencing approaches have made substantial advances in functional characterization of key genes involved in host/parasite interaction (45, 105–110). Combined with biochemical approaches such as immunoprecipitation and binding assays, the trimeric protein complex composed of FREPs (Fibrinogen-Related Proteins), TEP (ThioEster-containing Protein) and Biomphalysin appears to be crucial in the recognition and elimination of the parasites (111–113), but these different proteins are not the only pieces in the complex molecular interaction between *B. glabrata* and *S. mansoni* (103). Depending on the parasite and snail strains, several other genetic factors have been demonstrated to influence the probability of success in infection, known as compatibility polymorphism (44, 114–117) characterized by a reciprocal arms race between host immune weapons and parasite countermeasures. Concerning the snail’s immune response against other pathogens and intruders, functional data remain scattered despite several transcriptomic approaches conducted to decipher the signaling pathways and to identify key genes (43, 62, 118–120). However, the exposure of snail recombinant proteins or cell-free plasma against some pathogens revealed that some FREPs, biomphalysins, BgTEP1 and LBP/BPI are able to coat the microbial surface suggesting a major role in opsonization and/or in targeted lysis (66, 121–123). Faced with a wide variety of pathogens, the diversification of immune molecules appears to be the evolutionary path considered in invertebrates to mount a specific and effective defense response (99, 124–126). In *Biomphalaria*, some large repertoires of genes that encode immune receptors such as FREPs, Toll-like receptor (TLR) or TEPs have been reported with more than 30, 50 or 11 genes respectively (46, 63, 99, 127), while the number of effector genes identified may appear more restricted (128).

In this work, we report the characterization of 23 aerolysin-like genes in the snail *B. glabrata* after the initial discovery of biomphalysin 1 (42). The diversification of this family to 23 members likely occurred through multiple gene duplication events from at least one horizontally transferred ancestral gene. However, the phylogenetic analysis performed on aerolysin-related proteins revealed that biomphalysin sequences are not all distributed in a single monophyletic cluster. Since all biomphalysins except for biomphalysin 9 are distributed in a clade that includes other gastropods, we hypothesize that at least two HGT events potentially occurred without excluding the possibility of a different evolutionary history from a single horizontally acquired ancestral gene, which contributed to a high sequence diversity within the biomphalysin family (**Figure 2**). Considering the phylogenetic incongruence between biomphalysin 1 and aerolysin-related proteins and that biomphalysin genes are intron-less (42), it was proposed that biomphalysins were acquired from bacteria. This HGT of bacterial toxins was previously reported between species belonging to the different kingdoms (18, 35). The higher similarity in GC content and codon usage between the biomphalysins and other genes from *B. glabrata* compared to bacterium relatives, further support an ancient transfer followed by host domestication. HGT-derived genes gradually adapt the compositional traits of their host genome (GC content and codon usage) so that they are suitable for the host transcriptional and translational machineries, which lead to the functional integration into the host biological pathways (129, 130). Diversification of aerolysin-like families by gene duplication seems to be a shared feature among all invertebrates including toxins. This species-specific diversity of aerolysin-related toxins observed among different invertebrates strongly highlights that these acquired genes must confer an adaptive advantage for each recipient organism. In cnidaria, hydralysins are suspected to play a major role in protecting against predators or for killing prey (35, 37). In Gastropoda such as *Aplysia*, *Physella* or *Lymnaea*, the presence of toxin-related proteins could be used to escape predators as a defensive mechanism in *Physella acuta* and *Lymnaea stagnalis* (131–133). The success for aerolysin-related gene retention by various metazoan organisms could be linked to the absence of a similar preexisting metabolic network in the recipient organisms (134–136). Thus, the acquisition of new functions can easily be integrated into the recipient’s metabolome to provide an immediate benefit (137, 138) since they can be described as self-contained units (35). Aerolysin-related proteins are composed of two structurally independent lobes, with one involved in substrate interaction and the other in lytic activity (**Figure 4**). Notably, biomphalysin 1 has been shown to directly interact with the surface of *Schistosoma* sporocysts and its lytic activity was promoted by additional plasmatic factors (42). In the snail, all biomphalysins are expressed and all exhibited a tissue-specific pattern of expression. Some tissues such as the stomach expressed almost all biomphalysin genes while others, like the albumen gland, only expressed half of these genes (**Figure 7**). Together, this highlights that biomphalysin transcription and/or translation is successfully regulated by the recipient and suggests that each biomphalysin might have suffered a different selection pressure leading to different biological functions in the snail, including the sensing or elimination of pathogenic invaders.

The structure of the large lobe is highly conserved in all biomphalysins, which supports the conservation of their lytic activity (**Figure 5**). Conversely, structural predictions revealed high divergence between the (isolated) small lobes of biomphalysins and bacterial aerolysin (**Table S5**). While the small lobe of bacterial aerolysin is a carbohydrate-binding site (APT domain) (26), those of biomphalysins matched to different domains involved in moiety recognition or protein interaction. Interestingly, for about half of biomphalysins, the small lobe is structurally close to the Factor B (**Figure 6**), a key component of the activate complement of the alternative pathway in vertebrates (139). The interaction between Factor B and the cleaved complement component C3b to form and induce the stability of the C3 convertase is mediated by the Ba domain composed by three N-terminal CCP (Complement Control Protein) modules for which some small lobes of biomphalysins have close structural similarities (85, 140, 141). The CCP module, also known as the sushi domain, is harbored by many proteins involved in cell adhesion, in blood coagulation or in innate immunity (142). For example, human ApoH and Decay-Accelerating Factor (DAF), two CCP-containing proteins, have been shown to interact with pathogens such plasmodium and *E. coli*, respectively (143, 144). Together, this suggests that ligand-binding specificity of biomphalysins could be related to the structural nature of their small lobes. The interactome results showed that the repertoire of biomphalysins that bind to pathogens depends on the nature of the pathogen considered (66). A recent remarkable work based on recombinant proteins revealed the association between FREPs and BgTEP1 (113), thus confirming the previous results obtained by Mone and collaborators (111). In addition, biomphalysins can interact directly with the surface of *S. mansoni* without plasmatic cofactors (42) and have been shown to be associated with FREPs and BgTEP1 (113). This may suggest a double recognition of pathogens by at least FREP3 and biomphalysin (s) to induce an efficient lytic response since each protein component considered separately is unable to kill parasites (42, 113). Thus, we speculate that BgTEP1 and 2 might serve as the missing pieces of the puzzle between biomphalysin and FREP3, that are required to induce the lytic-pore formation. The biomphalysin-dependent recognition pathway can be achieved by single or multiple toxins, since tryptic peptides collected and analyzed by mass spectrometry identified in the present or previous study (113) could match to biomphalysins 1, 2, 3, 4 and 7. Since interactions between biomphalysins, C3-like proteins and FREPs are increasingly reported (111–113), their functional association could represent the proto-complement present in protostome invertebrates, and particularly in the Lophotrochozoa phylum. This proto-complement complex defined in a phylogenetic approach based on transcriptomic data from multiple invertebrates contains at least a C3 like-TEP, a serine protease C2/factor B and complement receptors (145, 146), to induce an activated complement cascade on the pathogen surface leading to the non-self opsonization and elimination. Some molecules of this proto-complement have been characterized from several mollusk species like Complement factor C2/B from Bivalvia such as *Ruditapes decussates* (147) or *Sinonovacula constricta* (148), C3-like factors from mussels (149) or clams (150), but few studies led to the characterization of the whole system (145, 151). While a diversity of C3-like proteins within the TEP superfamily has been reported (63, 99) as well as a large repertoire of carbohydrate-binding proteins such as FREPs (46, 152) and VIgL domain-containing proteins *in B. glabrata* (53, 112), no complement factor C2/B-like protein has yet been identified. As the small lobe from several biomphalysins is structurally close to the CCP domain of Factor B, we speculated that biomphalysins could be involved in the activation of the proto-complement system by binding with TEPs.

In conclusion, it appears that biomphalysin acquisition from a proto-aerolysin and expansion underline a neo- and sub-functionalization to supplement the snail immune system’s arsenal. The diverse biomphalysins appear to be key players in the immune repertoires as they harbor two essential functions: one in pathogen recognition and the second in pathogen elimination through their cytolytic activity. Therefore, a systematic molecular investigation on each biomphalysin member is expected to further unravel their specific functions in response to different pathogens.

## Data Availability Statement

The datasets presented in this study can be found in online repositories. The names of the repository/repositories and accession number(s) can be found in the article/**Supplementary Material**.

## Ethics Statement

The animal study was reviewed and approved by DRAAF Languedoc-Roussillon (Direction Régionale de l’Alimentation, de l’Agriculture et de la Forêt), Montpellier, France (Authorization #007083).

## Author Contributions

DD designed the research. SP, GT, RG, and BG substantially participated in conception and improvement of research. GT and RG performed the phylogenetic analysis. DD performed structural predictions. CC performed global analysis on promoter and search on duplication events on whole genome. SP and PP performed gene expression analysis. SP, AP, and GT performed interaction experiments. GT, SP, and DD led the manuscript writing. DL, PP, and ES annotate biomphalysin genes. All authors contributed to the article and approved the submitted version.

## Funding

This work was funded by ANR AeroSNAIL (number ANR-19-CE11-0016-01). DL received a PhD allocation (2017–2020) granted by the Fondation pour la Recherche Médicale (FRM). ES is supported by a PhD funding from the Region Occitanie (BioResist project) and the University of Perpignan Via Domitia graduate school ED305. This study is set within the framework of the “Laboratoires d’Excellences (LABEX)” TULIP (ANR-10-LABX-41). The funders had no role in study design, data collection and analysis, decision to publish or preparation of the manuscript.

## Conflict of Interest

The authors declare that the research was conducted in the absence of any commercial or financial relationships that could be construed as a potential conflict of interest.

## Supplementary Material

Supplementary Figure 1 Dot plot of the different biomphalysins and their surrounding genomic regions. The first row and column represent all the biomphalysins ordered from 1 to 23, followed by the (up to 20 kbp) genomic regions surrounding and including the biomphalysin. Blue circle, conserved structure between biomphalysins 5 and 6. Green circle, similarity region near biomphalysins 11 and 22.

Supplementary Figure 2 Sequence alignment and predicted topology of biomphalysin proteins. Multiple alignment of the biomphalysin proteins was performed using CLUSTALO. Conserved amino acid residues identified in 75% of all sequences are highlighted with a black box. Amino acids containing similar chemical properties are shown in a gray box. Key amino acids whose roles are described as important for oligomerization and lytic activity are indicated in white on a red background. The positions of secondary structure elements were predicted using the server PROMALS3D. α-helices and β-strands are indicated by “h” and “e”, respectively. The putative peptide signal indicated in brown were predicted on SignalP 4.1 server (153). The Transmembrane Domain boxed with a blue dashed line was predicted using the PRED –TMBB server. The signal peptide is in brown, the small lobe in red and the large lobe containing the aerolysin motif (IPR005830) in green. Structural domains according to the predicted structure of biomphalysin 1 are colored coded with domain 1 in red, domain 2 in green, domain 3 in blue and domain 4 in purple. The predicted residues involved in mannose binding are shown in a yellow box.

Supplementary Figure 3 TMD characterization in biomphalysin proteins. **(A)** Multiple alignment of the region surrounding the transmembrane domain of all biomphalysin proteins was performed using the HHpred server. The Transmembrane Domain boxed in blue was predicted using the PRED –TMBB server and confronted to the TMD of aerolysin protein (AFP82959) from *Aeromonas hydrophyla*. Hydrophylics (Ser and Thr) and Hydrophobics (Val, Ile, Leu and Ala) are shown in red and blue respectively. **(B)** A hydropathy plot of the predicted TMD from all biomphalysin proteins was done by the method of Kyte and DooLittle (1982). An alternation of hydrophilic and hydrophobic residues was observed for all predicted TMD. For example, TMD sequence of biomphalysin 2 is given in this plot. **(C)**. Structure of the TMD of biomphalysin 2 was predicted by the I Tasser server from the complete sequence of the protein. Close view on TMD was generated by PyMOLWin. Beta sheets represented by arrows forms a beta hairpin similar to the loop region in domain III of aerolysin toxin. All biomphalysins contains a transmembrane beta hairpin required to drive membrane insertion.

Supplementary Figure 4 Carbohydrate binding site prediction on biomphalysin proteins. **(A)** Surface representation of biomphalysin16 protein interacting with alpha-D mannose-6-phosphate (PDB: M6P). M6P are shown as spherical structures with gray carbon, phosphate and oxygen atoms. Ribbon representation of biomphalysin 16 was superimposed on a transparent surface map. **(B)** Close-up of the binding site. Alpha-D mannose-6-phosphate bound to the predicted pocket is located in the domain II of biomphalysin protein. The predicted residues involved in mannose binding are shown.

## Abbreviations

β-PFT, Beta-Pore Forming Toxin; CDS, Coding DNA Sequence; GPI, Glycosyl Phosphatidyl-Inositol; HGT, Horizontal Gene Transfer; M6P, Mannose-6-Phosphate; PCA, Principal Component Analysis; PDB, Protein Data Bank; TMD, Trans-Membrane Domain.
