# Supplementary figures and images for: New Insights Into Biomphalysin Gene Family Diversification in the Vector Snail Biomphalaria glabrata
Source: Front Immunol. 2021 Apr 1;12:635131. doi: 10.3389/fimmu.2021.635131 (PMC8047071; doi:10.3389/fimmu.2021.635131)

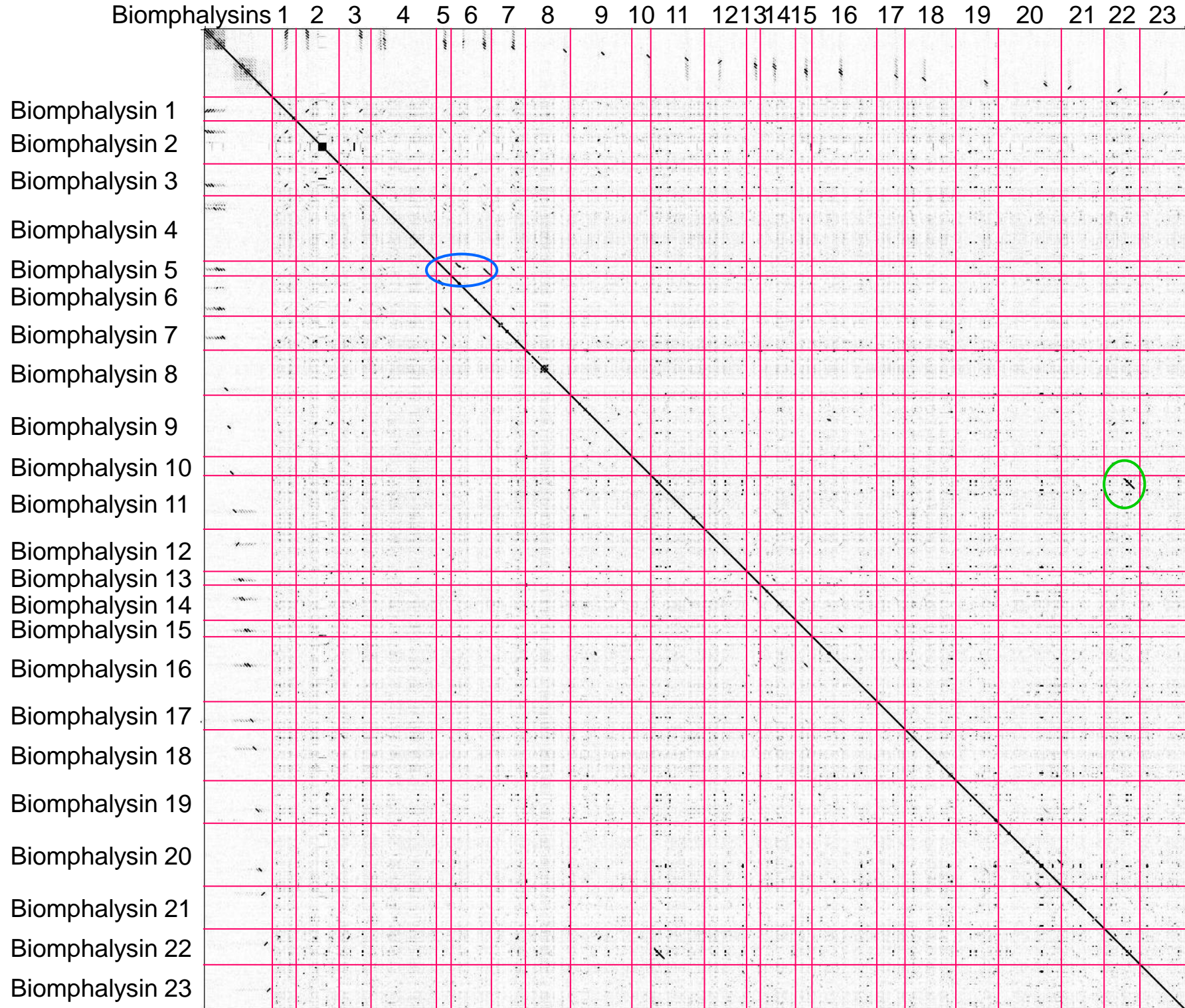

Supplement: Supplementary Figure 1 — Dot plot of the different biomphalysins and their surrounding genomic regions. The first row and column represent all the biomphalysins ordered from 1 to 23, followed by the (up to 20 kbp) genomic regions surrounding and including the biomphalysin. Blue circle, conserved structure between biomphalysins 5 and 6. Green circle, similarity region near biomphalysins 11 and 22. [file Image_1.pdf]

A

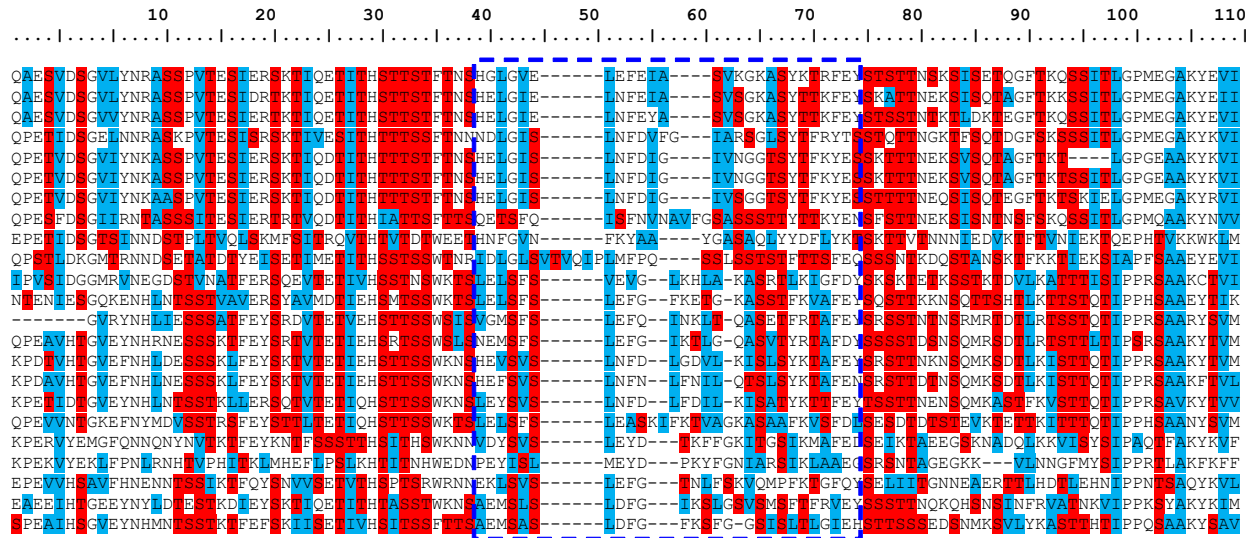

B

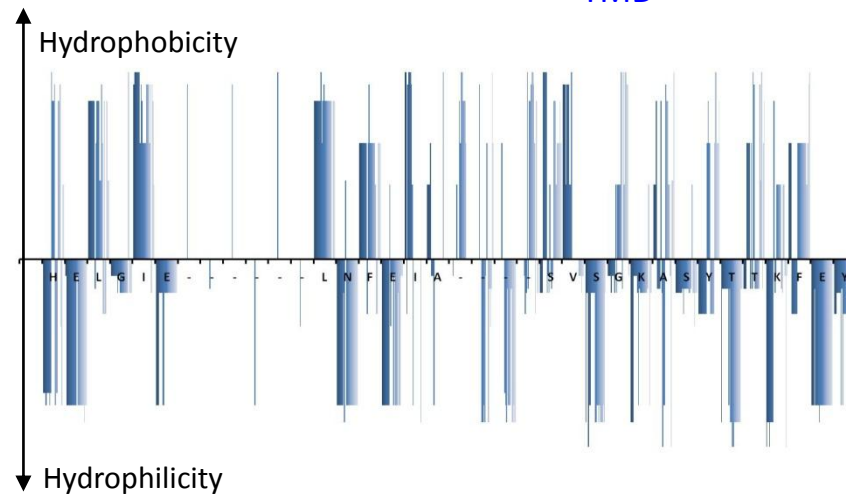

C

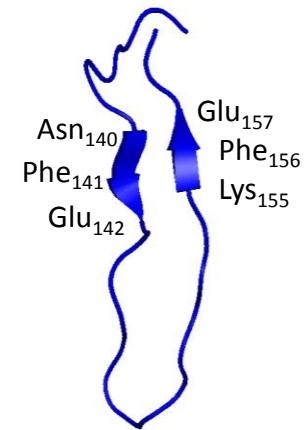

Supplement: Supplementary Figure 3 — TMD characterization in biomphalysin proteins. (A) Multiple alignment of the region surrounding the transmembrane domain of all biomphalysin proteins was performed using the HHpred server. The Transmembrane Domain boxed in blue was predicted using the PRED –TMBB server and confronted to the TMD of aerolysin protein (AFP82959) from Aeromonas hydrophyla. Hydrophylics (Ser and Thr) and Hydrophobics (Val, Ile, Leu and Ala) are shown in red and blue respectively. (B) A hydropathy plot of the predicted TMD from all biomphalysin proteins was done by the method of Kyte and DooLittle (1982). An alternation of hydrophilic and hydrophobic residues was observed for all predicted TMD. For example, TMD sequence of biomphalysin 2 is given in this plot. (C). Structure of the TMD of biomphalysin 2 was predicted by the I Tasser server from the complete sequence of the protein. Close view on TMD was generated by PyMOLWin. Beta sheets represented by arrows forms a beta hairpin similar to the loop region in domain III of aerolysin toxin. All biomphalysins contains a transmembrane beta hairpin required to drive membrane insertion. [file Image_3.pdf]

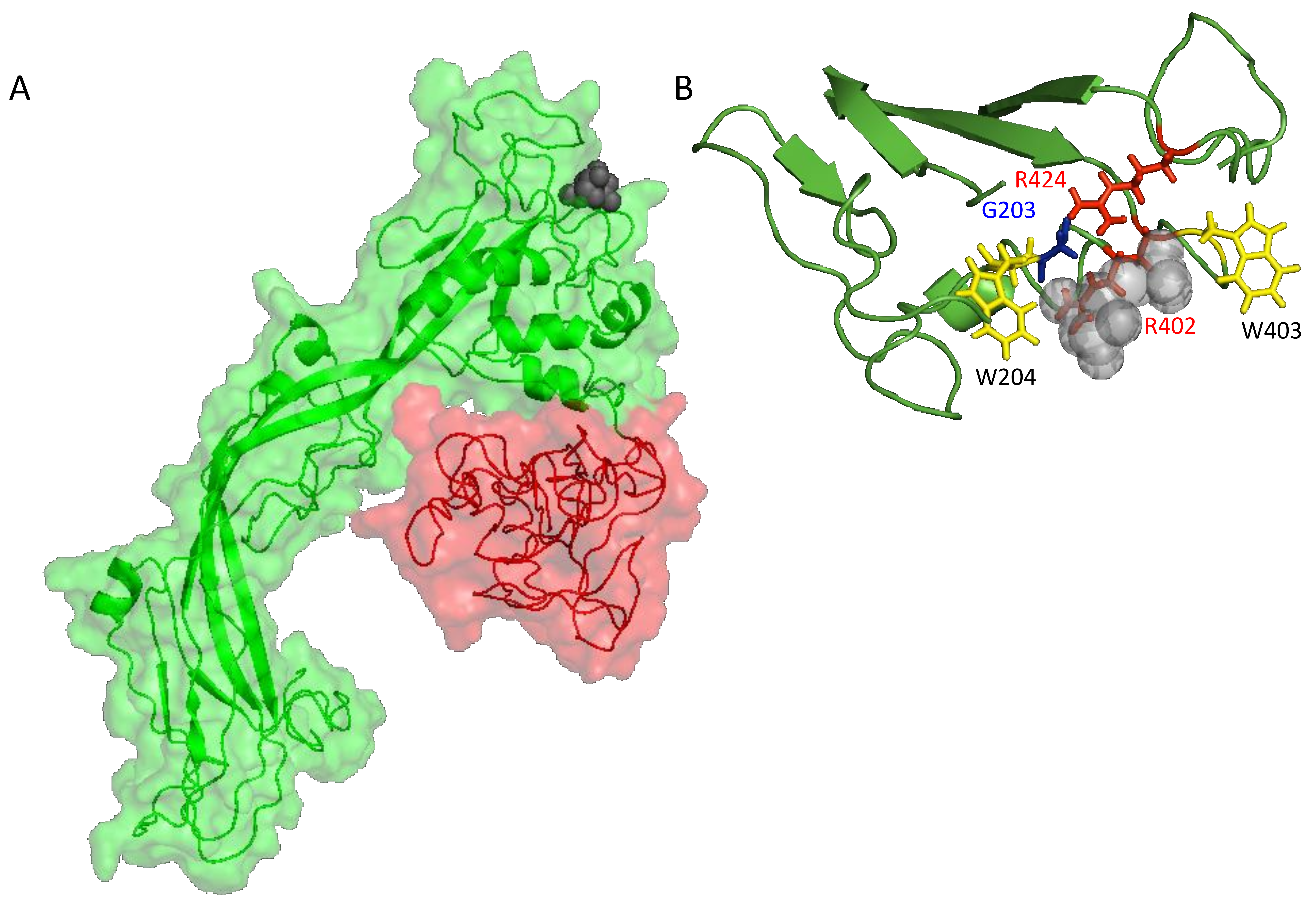

Supplement: Supplementary Figure 4 — Carbohydrate binding site prediction on biomphalysin proteins. (A) Surface representation of biomphalysin16 protein interacting with alpha-D mannose-6-phosphate (PDB: M6P). M6P are shown as spherical structures with gray carbon, phosphate and oxygen atoms. Ribbon representation of biomphalysin 16 was superimposed on a transparent surface map. (B) Close-up of the binding site. Alpha-D mannose-6-phosphate bound to the predicted pocket is located in the domain II of biomphalysin protein. The predicted residues involved in mannose binding are shown. [file Image_4.tif]
