# Supplementary material for: New Insights Into Biomphalysin Gene Family Diversification in the Vector Snail Biomphalaria glabrata
Source: Front Immunol. 2021 Apr 1;12:635131. doi: 10.3389/fimmu.2021.635131 (PMC8047071; doi:10.3389/fimmu.2021.635131)
Supplement: Supplementary Figure 2 — Sequence alignment and predicted topology of biomphalysin proteins. Multiple alignment of the biomphalysin proteins was performed using CLUSTALO. Conserved amino acid residues identified in 75% of all sequences are highlighted with a black box. Amino acids containing similar chemical properties are shown in a gray box. Key amino acids whose roles are described as important for oligomerization and lytic activity are indicated in white on a red background. The positions of secondary structure elements were predicted using the server PROMALS3D. α-helices and β-strands are indicated by “h” and “e”, respectively. The putative peptide signal indicated in brown were predicted on SignalP 4.1 server (153). The Transmembrane Domain boxed with a blue dashed line was predicted using the PRED –TMBB server. The signal peptide is in brown, the small lobe in red and the large lobe containing the aerolysin motif (IPR005830) in green. Structural domains according to the predicted structure of biomphalysin 1 are colored coded with domain 1 in red, domain 2 in green, domain 3 in blue and domain 4 in purple. The predicted residues involved in mannose binding are shown in a yellow box. [file Image_2.pdf]

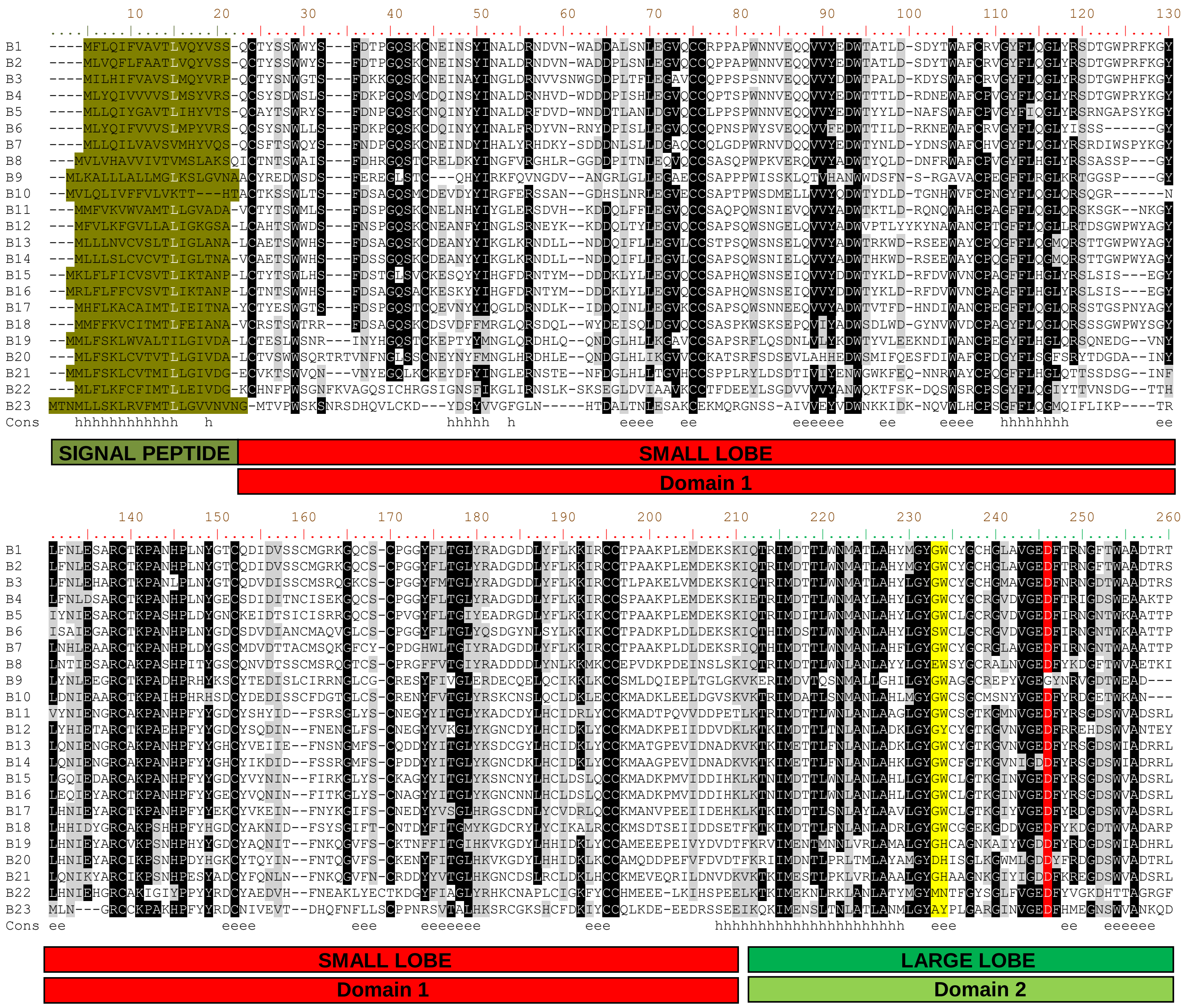
SIGNAL PEPTIDE
SMALL LOBE
Domain 1
SMALL LOBE
Domain 1
LARGE LOBE
Domain 2

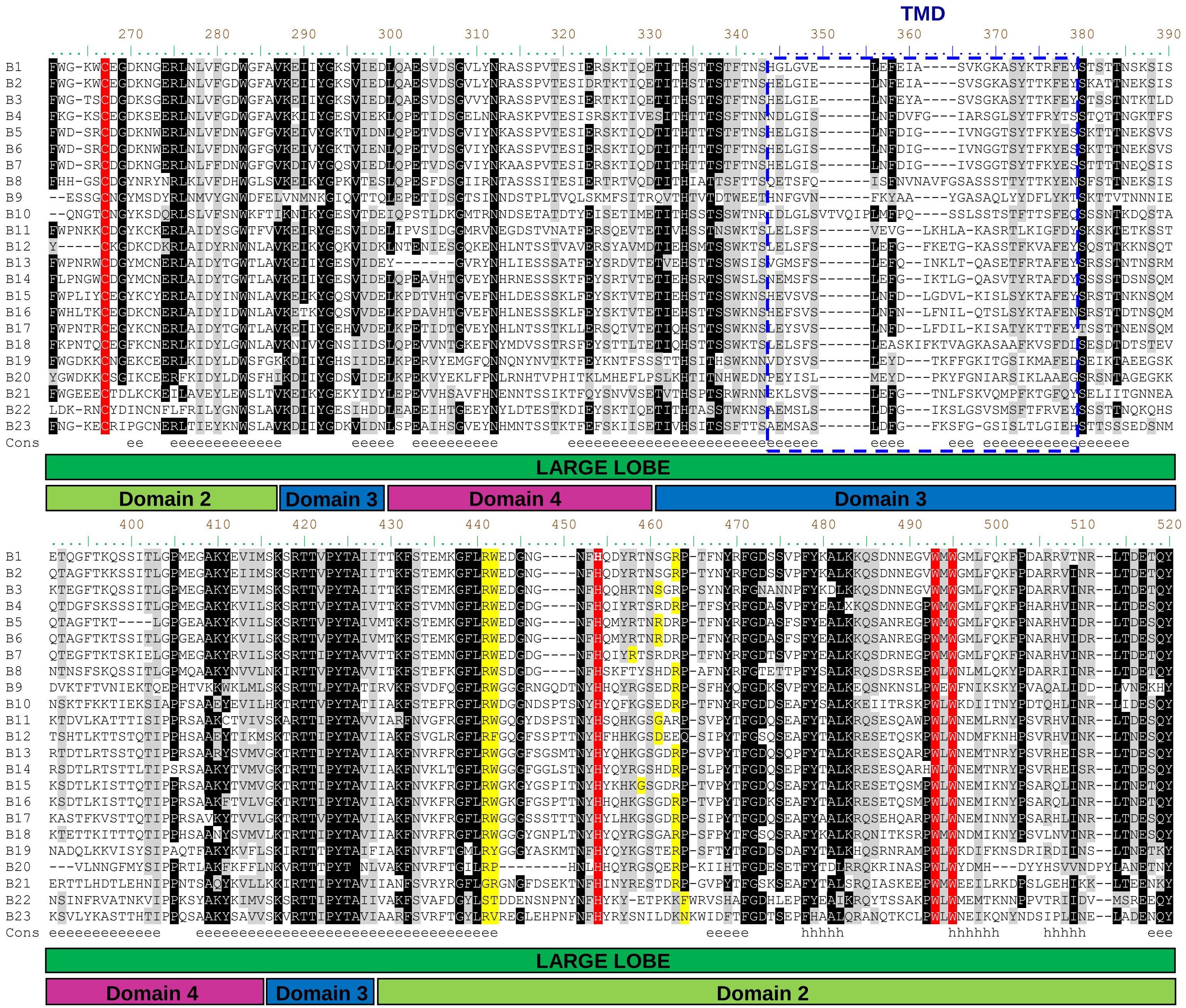

TMD
LARGE LOBE
Domain 2
Domain 3
Domain 4
Domain 3
LARGE LOBE
Domain 4
Domain 3
Domain 2

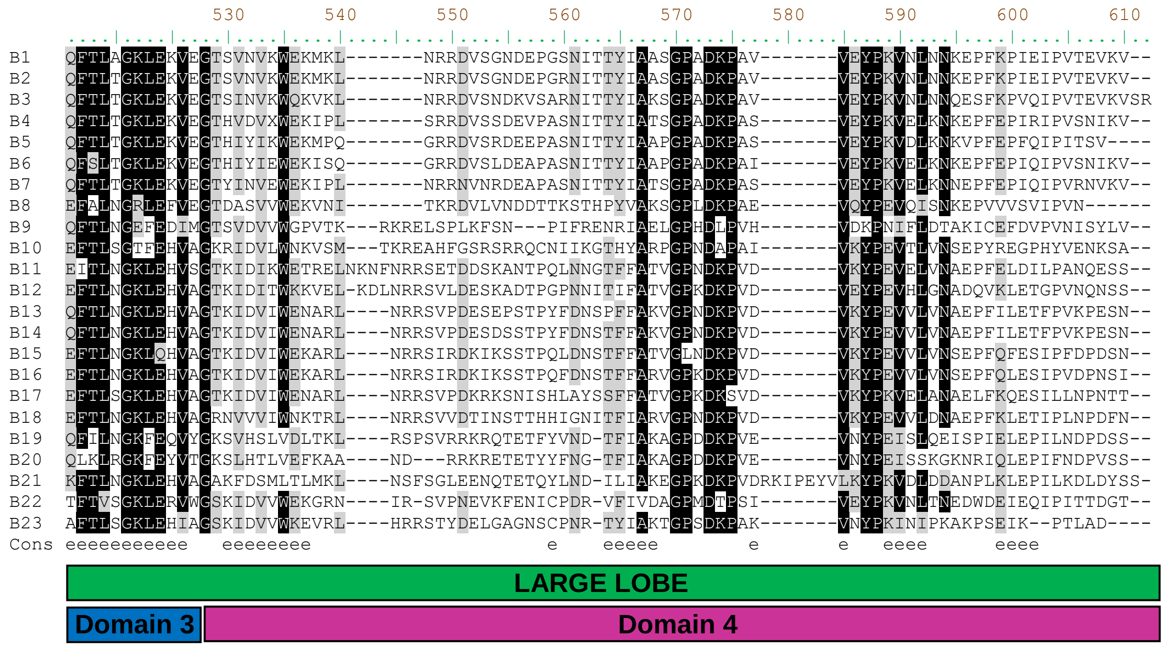

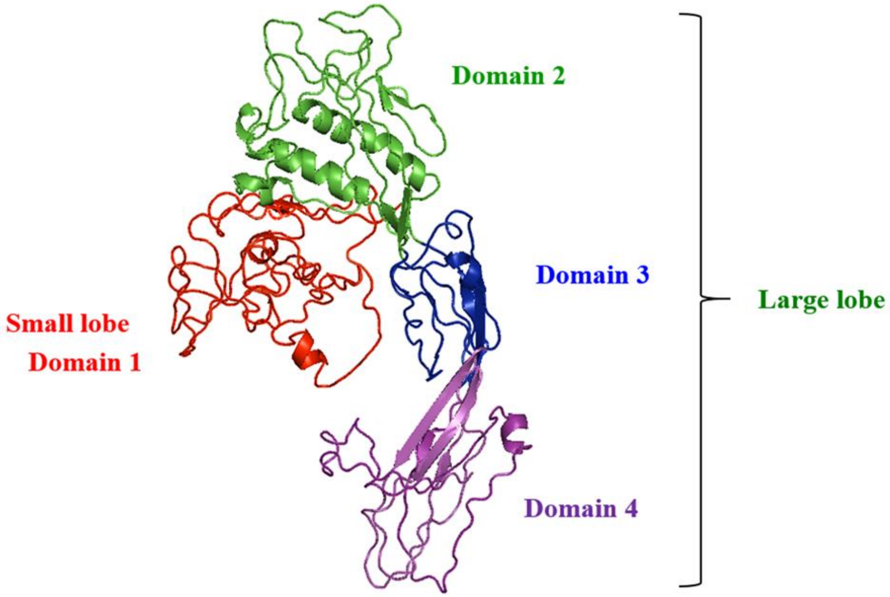

Biomphalysin 1
